# Transcription factor-mediated germ cell induction in rats reveals ETV4 cooperates with germline specifiers

**DOI:** 10.1016/j.stemcr.2025.102599

**Published:** 2025-08-12

**Authors:** Mami Oikawa, Hiroki Kojima, Hisato Kobayashi, Kenyu Iwatsuki, Hijiri Saito, Makoto Sanbo, Kazumi Nishioka, Tomoyuki Yamaguchi, Takuya Yamamoto, Kazuki Kurimoto, Masumi Hirabayashi, Toshihiro Kobayashi

**Affiliations:** 1 Division of Mammalian Embryology, Center for Stem Cell Biology and Regenerative Medicine, The Institute of Medical Science, The University of Tokyo, Minato-ku, Tokyo 108-8639, Japan; 2 Laboratory of Regenerative Medicine, Tokyo University of Pharmacy and Life Science, Hachioji, Tokyo 192-0392, Japan; 3 Department of Embryology, Nara Medical University, Kashihara, Nara 634-8521, Japan; 4 Department of Medical Genome Science, Dokkyo Medical University, Mibu, Tochigi 321-0293, Japan; 5 Division of Mammalian Embryogenesis, Department of Homeostatic Regulation, National Institute for Physiological Sciences, Okazaki, Aichi 444-8787, Japan; 6 Center for iPS Cell Research and Application, Kyoto University, Sakyo-ku, Kyoto 606-8507, Japan; 7 Institute for the Advanced Study of Human Biology, Kyoto University, Sakyo-ku, Kyoto 606-8501, Japan; 8 Medical-risk Avoidance based on iPS Cells Team, RIKEN Center for Advanced Intelligence Project, Sakyo-ku, Kyoto 606-8507, Japan; 9 The Graduate University of Advanced Studies, Okazaki, Aichi 444-8787, Japan

**Keywords:** primordial germ cell, rat, pluripotent stem cell

## Abstract

The specification of primordial germ cells (PGCs) marks a crucial branchpoint in early embryonic development. Studying the molecular mechanisms governing this process is crucial for understanding reproduction and evolution. Here, we identify transcription factors essential for PGC specification in rats using an *in vitro* system to induce PGC-like cells (PGCLCs) from pluripotent cells. Overexpression of *Tbxt*, a key mesodermal factor activating the germ cell program in epiblast-like cells, induces functional rat PGCLCs, similar to mice. However, unlike in mice, overexpression of the PGC specifiers (*Prdm14*, *Blimp1*, and *Ap2γ*) alone is not sufficient in rats; additional Activin and WNT signals are necessary for PGCLC induction. Through a candidate screen, we identified the transcription factor *Etv4* acting cooperatively with the three PGC specifiers. Our study provides insight into the mechanism behind germline segregation in mammals and underscores the importance of using the rat model in addition to mice.

## Highlights

• Overexpression of *Tbxt* in rEpiLCs can induce functional rPGCLCs without exogenous BMP4
• Unlike in mice, *Prdm14/Blimp1/Ap2γ* requires Activin/WNT signals for PGCLC induction in rats
• *Etv4,* a target of Activin/WNT signals, cooperates with *Prdm14/Blimp1/Ap2γ* to induce rPGCLCs

## Graphical abstract

**Figure:**
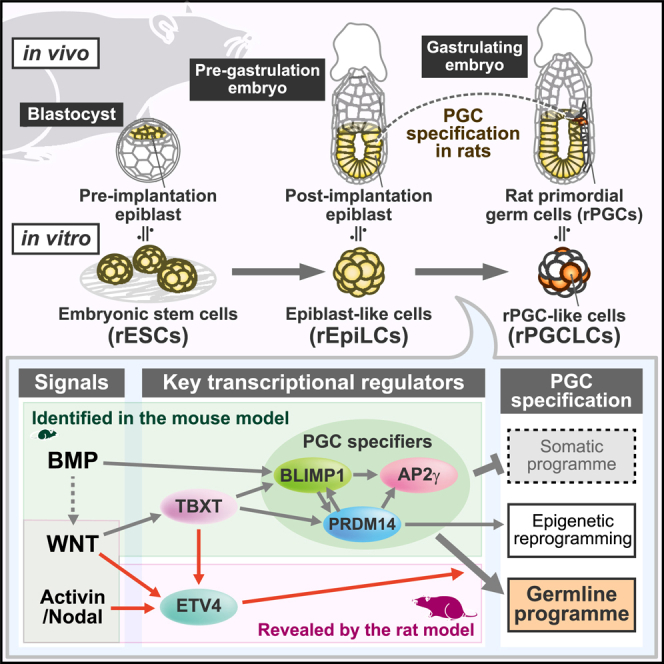

## Teaser

Oikawa et al. explored key transcription factors for PGC specification in rats. Using an *in vitro* rPGCLC induction system, they found that overexpression of *Tbxt* in rEpiLCs produced functional rPGCLCs without requiring exogenous BMP4. In addition, they discovered that *Etv4*, a downstream target of Activin/WNT signals, cooperates with *Prdm14/Blimp1/Ap2γ* for rPGCLC induction. This study highlights the value of the rat model alongside mice for elucidating fundamental developmental pathways.

## Introduction

The germline, a unique cell lineage capable of transmitting genetic information across generations, is one of the first lineages to segregate from pluripotent cells during early mammalian development (T. Kobayashi and Surani, 2018). Studies on the extrinsic and intrinsic factors that specify primordial germ cells (PGCs), the founder cells for sperm and eggs, offer fundamental insights into cell fate decisions. In mice, the proximal posterior epiblast is directed toward PGC fate through the successive action of extrinsic wingless (WNT) and bone morphogenetic protein (BMP) signals, as demonstrated by knockout (KO) studies and *ex vivo* epiblast cultures (Ohinata et al., 2009; Ying et al., 2001). Understanding the intrinsic transcriptional program has been technically challenging due to the small number of specified PGCs (<40). However, this limitation has been addressed through recent advancements in the development of *in vitro* systems inducing PGC-like cells (PGCLCs) from pluripotent stem cells (PSCs). In this system, the transition from naive mouse PSCs to formative epiblast-like cells (EpiLCs) recapitulates post-implantation epiblast development. Subsequently, the formation of aggregates in the presence of BMP4, a cytokine critical for PGC fate, efficiently induces PGCLCs (Hayashi et al., 2011).

Using this robust *in vitro* system, Nakaki et al., demonstrated that overexpression of three germ cell specifiers, *Prdm14*, *Blimp1* (also known as *Prdm1*), and *Ap2γ* (also known as *Tfap2c*) (Ohinata et al., 2005; Weber et al., 2010; Yamaji et al., 2008), in mouse EpiLCs (mEpiLCs) efficiently induces mouse PGCLCs (mPGCLCs) without the need for external BMP (Nakaki et al., 2013). Among the three PGC specifiers, *Prdm14* plays a central role in inducing the germline program as well as epigenetic reprogramming in mice (Nakaki et al., 2013; Seki, 2018). While, *Tbxt* (*Brachyury, T*), a mesodermal factor, can also direct epiblast toward PGC fate by activating the PGC specifiers (Aramaki et al., 2013). These *in vitro* systems have also been applied to other mammals, including human PSCs to investigate the molecular mechanisms underlying PGC specification (Kobayashi et al., 2017; Kojima et al., 2017; Kojima et al., 2021; Tang et al., 2022).

Recently, we successfully established an *in vitro* system to induce PGCLCs in rats, a widely used experimental rodent animal model alongside mice (Oikawa et al., 2022). We found that, unlike adherent monolayer cultures in mice, rat EpiLCs induced from pluripotent embryonic stem cells (rESCs) require the formation of spherical aggregates for PGCLC induction. Notably, rat PGCLCs (rPGCLCs) are fully functional and able to complete spermatogenesis upon transplantation into seminiferous tubules of germ cell-free *Prdm14* KO rats (Kobayashi et al., 2020, 2021). Furthermore, the rPGCLC-derived spermatids/sperm contribute to the birth of offspring, a result that had only been previously reported for mice.

Utilizing this reliable *in vitro* system and *in vivo* stringent assay, here, we investigate the transcriptional factor(s) (TF(s)) driving PGC fate in rats. We find that TFs important for PGC specification found in mice are well conserved in rats. However, supplementation of Activin and WNT signals is necessary to induce functional PGCLCs by the three PGC specifiers (*Prdm14*, *Blimp1*, *and Ap2γ*). Furthermore, we identify *Etv4*, a potential downstream target gene of these signals, which acts cooperatively to induce PGC fate.

## Results

### *Tbxt* induces functional PGCLCs in rats

**Figure 1:**
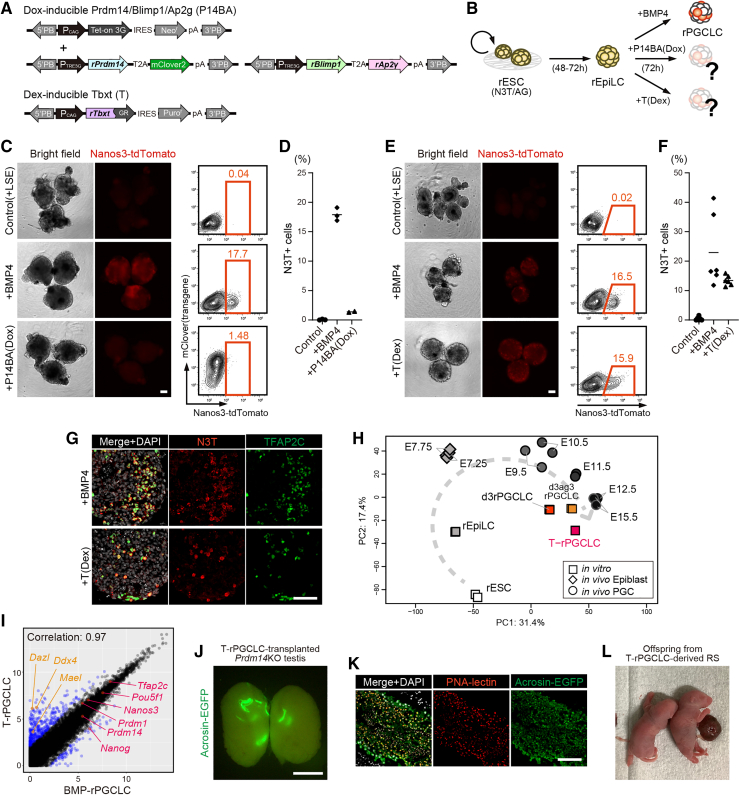
*Tbxt* but not *Prdm14/Blimp1/Ap2γ* can induce functional rPGCLC in the absence of BMP4 (A) Gene-inducible system in this study; Dox-inducible *rPrdm14-T2A-mClover2*, *rBlimp1-T2A-rAp2γ*, and Dex-inducible *rTbxt*. (B) Experimental design of transcription factor(s)-mediated rPGCLC induction. (C) Images and FACS patterns of day 3 rPGCLCs induced by only LSE, LSE plus BMP4, and LSE plus P14BA by adding Dox. Scale bar is 100 μm. (D) Dot plot showing percentage of Nanos3-tdTomato (N3T)-positive cells in Figure 1C (*n* = 2–3 biologically independent experiments). (E) Images and FACS patterns of day 3 rPGCLCs induced by only LSE, LSE plus BMP4, and LSE plus T by adding Dex. Scale bar is 100 μm. (F) Dot plot showing percentage of Nanos3-tdTomato (N3T)-positive cells in Figure 1E (*n* = 6 biologically independent experiments). (G) IF images of day 3 rPGCLCs induced by BMP4 or T. Scale bar is 100 μm. (H) PCA showing the position of T-rPGCLCs within the developmental trajectory, integrating both *in vivo* and *in vitro* datasets. All transcriptome samples were obtained from 2 biologically independent experiments. The same applies below. (I) Scatterplot showing correlation between BMP- and T-induced rPGCLCs. (J) Testis 12 weeks after transplantation of T-rPGCLCs. Some seminiferous tubules positive for GFP indicate spermatogenesis originated from T-rPGCLCs. Scale bar is 5 mm. (K) IF images of GFP-positive seminiferous tubules in Figure 1J. Scale bar is 100 μm. (L) Offspring obtained by injection of T-rPGCLC-derived round spermatids into unfertilized rat oocytes. See also Figure S1 and Tables S1 and S2.

**Figure 2:**
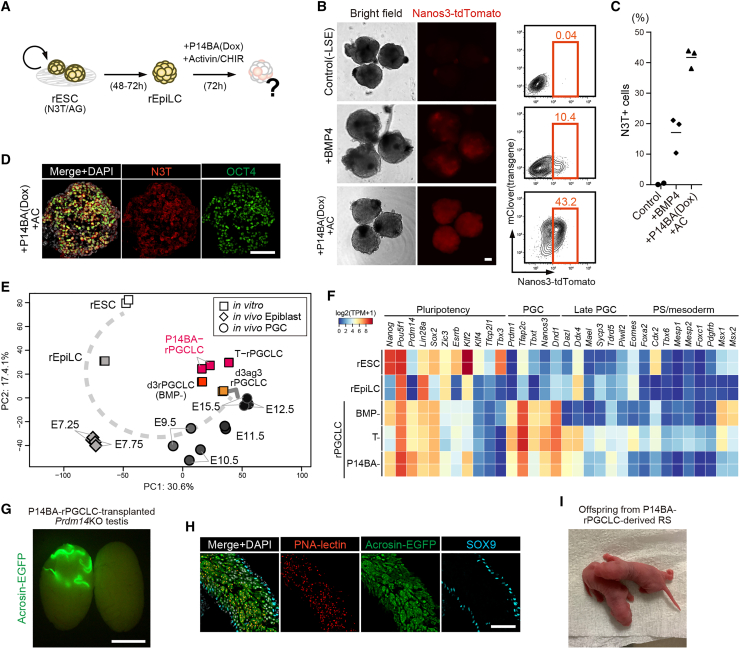
Supplementation of Activin and WNT signals allows *Prdm14/Blmip1/Ap2γ* to induce functional rPGCLC in the absence of BMP4 (A) Schematics of P14BA-mediated rPGCLC induction with supplementation of Activin-A and CHIR99021 (AC). (B) Images and FACS patterns of day 3 rPGCLCs induced by only LSE, LSE plus BMP4, and LSE plus P14BA by adding Dox with AC. Scale bar is 100 μm. (C) Dot plot showing percentage of Nanos3-tdTomato (N3T)-positive cells in Figure 2B (*n* = 2–3 biologically independent experiments). (D) IF images of day 3 rPGCLCs induced by P14BA with AC. Scale bar is 100 μm. (E) PCA showing the position of P14BA-rPGCLCs within the developmental trajectory, integrating both *in vivo* and *in vitro* datasets. (F) Heatmap of representative gene expression of indicated samples. (G) Testis 12 weeks after transplantation of P14BA-rPGCLCs. Scale bar is 5 mm. (H) IF images of GFP-positive seminiferous tubules in Figure 2G. Scale bar is 100 μm. (I) Offspring obtained by injection of P14BA-rPGCLC-derived round spermatids into unfertilized rat oocytes. See also Figures S2 and S3 and Tables S1 and S2.

To verify whether the key TFs directing mouse PGC fate are conserved in rats, we first tested if rEpiLCs are competent to induce rPGCLCs in response to exogenous TF(s) in the absence of BMP4. Based on previous reports from the mouse model (Aramaki et al., 2013; Nakaki et al., 2013), doxycycline (Dox)-inducible *Prdm14-T2A-mClover2* (P14) alone, P14 together with *Blimp1-T2A-Ap2γ* (P1A), or dexamethasone (Dex)-inducible *Tbxt* (T) were introduced into rESCs (Figure 1A). For rESCs, we used two independent double reporter male rESC lines (rN3TAG#2 and #3) harboring both PGC-specific *Nanos3-T2A-tdTomato* (N3T) and spermatogenic cell-specific *Acrosin-EGFP* (AG) (Oikawa et al., 2022). Unless otherwise specified, the representative data shown in the figures were obtained using rN3TAG#2. P14-, P14BA- or T-inducible N3T/AG-rESCs were differentiated into rEpiLC aggregates for 48–72 h; the aggregates were then transferred into N2B27 + 5% knockout serum replacement (KSR) medium containing cytokines (rat leukemia inhibitory factor [LIF], mouse stem cell factor [mSCF], and mouse epidermal growth factor [mEGF], hereafter, LSE) with or without Dox/Dex (Figure 1B). In contrast to the previous data obtained from the mouse model, neither P14 nor P14BA induced N3T-positive rPGCLCs (Figures 1C, 1D, S1A, and S1B). Instead, consistent with mouse data, exogenous T successfully induced N3T expression at nearly similar efficiency and intensity as BMP4-induced rPGCLCs (hereafter, BMP-rPGCLCs) (Figures 1E and 1F). We also found that T was sufficient to induce N3T-positive rPGCLCs even in the absence of LSE cytokines, although with a lower efficiency (Figures S1C and S1D). Immunofluorescence (IF) analysis revealed that T-induced N3T-positive cells express the pluripotency and germ cell markers, TFAP2C and OCT4 (Figures 1G and S1E). We next analyzed the gene expression profiles of T-induced rPGCLCs (hereafter denoted as T-rPGCLC) by RNA sequencing (RNA-seq) and compared the transcriptomic data with *in vivo* rPGC and *in vitro* rPGCLC from our previous datasets (Kobayashi et al., 2020; Oikawa et al., 2022). Hierarchical clustering, correlation heatmap, and principal-component analysis (PCA) revealed that the transcriptome of T-rPGCLCs is highly similar to that of BMP-rPGCLCs, closely corresponding to embryonic day (E)9.5–12.5 *in vivo* rat PGCs (Figures 1H, 1I, S1F, and S1G). Both T-rPGCLCs and BMP-rPGCLCs express representative pan-PGC markers (*Prdm14*, *Tfap2c*, *Blimp1*, and *Nanos3*) and pluripotency markers (*Pou5f1* and *Sox2*). In addition, T-rPGCLCs showed modest upregulation of some primitive streak (PS)/mesoderm markers (*Eomes*, *Cdx2*, *Tbx6*, *Mesp1*, *Mesp2*, *Foxc1*, and *Pdgfrb*) as well as late PGC markers (*Dazl*, *Ddx4*, and *Mael*) (Figures 1I and 2F). To confirm the function of T-rPGCLCs, we tested their capacity to undergo spermatogenesis after transplantation into the seminiferous tubules of *Prdm14* KO rats. After 9–12 weeks of transplantation, we observed some seminiferous tubules filled with AG-positive cells (Figure 1J; Table S1). IF analysis revealed the presence of AG and peanut agglutinin (PNA)-lectin double-positive round spermatids and sperm, indicating successful spermatogenesis originating from T-rPGCLCs (Figure 1K). Finally, we performed round spermatid injection (ROSI) using T-rPGCLC-derived spermatids and found that AG-positive round spermatids are capable of producing viable offspring (Figure 1L; Table S2). Taken together, we clearly demonstrate that T-rPGCLCs, induced in the absence of external BMP signals, are fully functional and equivalent to BMP-rPGCLCs.

In mice, *Tbxt* is known to directly activate the expression of the PGC specifiers such as *Prdm14* and *Blimp1*, as well as mesodermal genes (Aramaki et al., 2013). Indeed, in rats, exogenous activation of *Tbxt* in rEpiLCs similarly led to a rapid (∼16 h) upregulation of *Prdm14* and *Blimp1* as well as endogenous *Tbxt* and the mesodermal gene *Cdx2* (Figure S1H). Since extraembryonic mesodermal cells also express BMP4 (Lawson et al., 1999), we added the BMP signaling inhibitor LDN-193189 (LDN) during BMP- or T-rPGCLC induction to eliminate any indirect effects from mesoderm-derived BMP. As expected, BMP-induced rPGCLC induction was almost completely inhibited in the presence of LDN (Figure S1I). By contrast, overexpression of *Tbxt* in the presence of LDN reduced (∼50%), but did not completely abolish rPGCLC induction (Figure S1I). This suggests that T-rPGCLCs comprises cells directly induced by *Tbxt* and others indirectly induced via *Tbxt*-mediated mesodermal BMP4 signaling.

### Induction of rat PGCLCs by *Prdm14/Blimp1/Ap2*γ requires concomitant activation of signals for PS fate

We then asked why P14 or P14BA, downstream targets of *Tbxt*, are unable to initiate the germline program in rats, unlike in mice (Nakaki et al., 2013). Since rodent PGCs are specified in the posterior epiblast, where PS formation occurs, we hypothesize that activation of PS signals, combined with the enforced expression of these PGC specifiers, might induce rPGCLCs. In mice, Activin and WNT signals are essential for the induction of PS fate and for PGC specification (Aramaki et al., 2013; Gadue et al., 2006; Kobayashi et al., 2017; Loh et al., 2014; Ohinata et al., 2009). We confirmed that adding inhibitors against both canonical WNT signal and WNT secretion (XAV939, IWP- 2) or an inhibitor of transforming growth factor β (TGF-β) Type I Receptor/ALK5 (SB-431542) nearly completely abrogated BMP-rPGCLC induction (Figure S2A), suggesting that the role of these signals in PGC specification are well conserved between mouse and rat. To activate the signaling pathway, we used Activin-A and CHIR99021 (AC), an inhibitor of glycogen synthase kinase-3 (GSK3) to activate canonical WNT pathways (Figure 2A). In the presence of AC, overexpression of P14BA efficiently induced N3T-positive rPGCLCs (P14BA-rPGCLCs; Figures 2B–2D), even in the absence of LSE cytokines. We confirmed that efficient induction of rPGCLCs, in the presence of AC, also can be achieved using a polycistronic vector (Figures S2B–S2D). This result is consistent in an independent cell line (rN3TAG#3) carrying the same transgenes (Figures S2E and S2F). Adding either Activin-A or CHIR99021 individually had a subtle effect on rPGCLC induction (Figures S2C and S2D), suggesting their synergistic role in enhancing rPGCLC induction, similar to PS fate induction as shown previously (Gadue et al., 2006). P14 or BA alone could also induce N3T-positive cells in the presence of AC, although with lower efficiency than P14BA (Figures S2G–S2J), suggesting a synergistic effect of the tripartite PGC specifiers (Magnusdottir et al., 2013; Nakaki et al., 2013). The transcriptome of P14BA-rPGCLCs is highly similar to that of BMP- and T-rPGCLCs (Figures 2E, S3A, and S3B). Like T-rPGCLCs, P14BA-rPGCLCs exhibit an overall similar gene expression pattern comparable to BMP-rPGCLCs, except for a modest upregulation of late PGC markers (Figure 2F). While T-rPGCLCs show a slight upregulation of some PS/mesodermal genes, P14BA-rPGCLC do not show this upregulation and instead further downregulate certain genes (*Cdx2*, *Msx1*, and *Msx2*) (Figure 2F). Thus, despite AC supplementation, which can induce PS fate, the subsequent or concomitant activation of the three PGC specifiers suppresses the somatic program, as shown in mice (Kurimoto et al., 2008). Functionally, P14BA-rPGCLCs transplanted into *Prdm14* KO neonatal testis were able to reconstitute spermatogenesis (Figures 2G and 2H; Table S1). Furthermore, isolated P14BA-rPGCLC-derived round spermatids contributed to the generation of viable offspring via ROSI (Figure 2I; Table S2), suggesting that P14BA-rPGCLCs are fully functional, similar to BMP-rPGCLCs. Overall, AC supplementation, which drives rEpiLCs toward a PS fate, promotes the induction of functional rPGCLCs through the activation of P14BA.

### *Etv4*, identified as a downstream target of Activin and WNT signals, acts cooperatively with PGC specifiers

**Figure 3:**
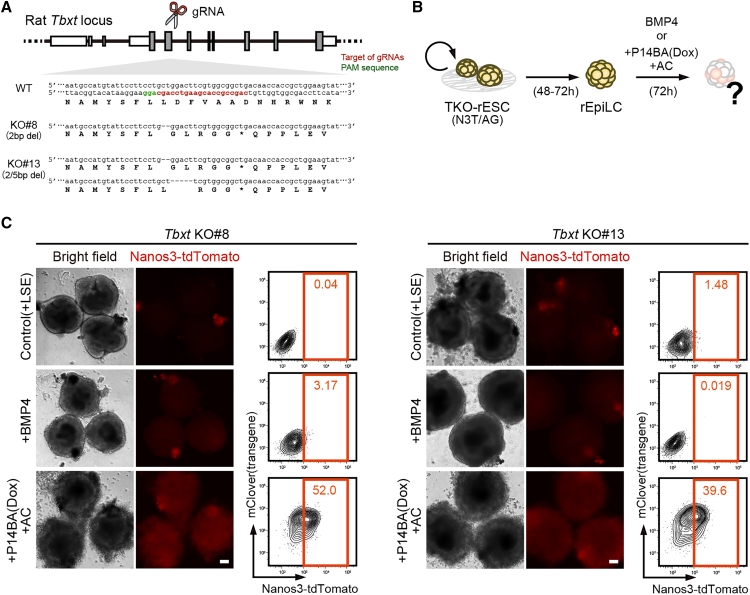
*Tbxt* is not necessary for induction of rPGCLCs by *Prdm14/Blmip1/Ap2γ* with Activin and WNT signals (A) Strategy to generate TKO rESC lines by CRISPR-Cas9 system and their genotype (#8 and #13). (B) Experimental design of induction of rPGCLC from TKO rESC lines. (C) Images and FACS patterns of TKO day 3 rPGCLCs induced by only LSE, LSE plus BMP4, and LSE plus P14BA by adding Dox with AC. Results of 2 independent clones generated in Figure 3A are shown. Scale bar is 100 μm.

Since AC is known to upregulate PS genes, including *Tbxt*, we speculated that endogenous *Tbxt* activated by AC, together with exogenous P14BA led to the successful induction of rPGCLCs. To test this hypothesis, we disrupted *Tbxt* in P14BA-inducible rESCs (TKO), and examined whether P14BA can induce rPGCLCs in the presence of AC without *Tbxt* (Figures 3A and 3B). Consistent with previous observations in mice, two independent lines (#8, #13) of TKO rESCs formed rEpiLC normally, but failed to induce rPGCLCs in response to BMP signaling (Figure 3C). Unexpectedly, however, P14BA with AC was able to induce rPGCLCs with nearly similar efficiency as the WT control, even in the absence of *Tbxt* (Figure 3C). Thus, T-independent P14BA-rPGCLC induction implies the existence of other factor(s) that may act as downstream targets of AC, facilitating rPGCLC induction.

**Figure 4:**
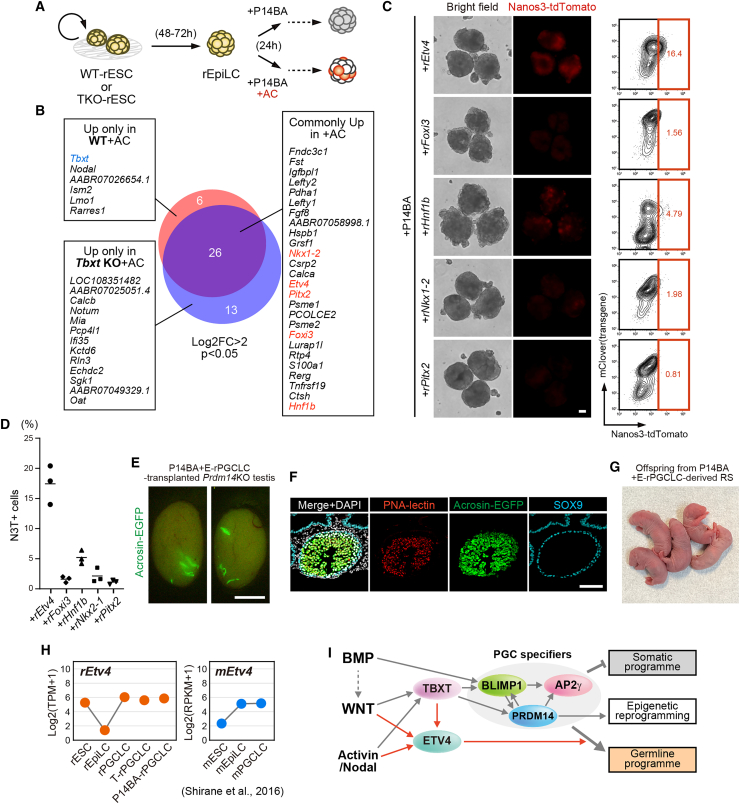
Candidate screening identifies *Etv4* acting cooperatively with *Prdm14/Blimp1/Ap2γ* to induce functional rPGCLCs (A) Schematics to identify downstream target(s) of Activin and WNT signals during rPGCLC induction. At 24 h post-induction with Dox, with or without AC, cells were collected and subjected to RNA-seq analysis. (B) Venn diagram showing genes upregulated in the presence of AC. Twenty six genes are commonly upregulated between WT and TKO. (C) Images and FACS patterns of day 3 rPGCLCs induced by P14BA together with candidate transcription factors. Scale bar is 100 μm. (D) Dot plot showing percentage of Nanos3-tdTomato (N3T)-positive cells in Figure 4C (*n* = 3 biologically independent experiments). (E) Testis 12 weeks after transplantation of P14BA+E-rPGCLCs. Scale bar is 5 mm. (F) IF images of GFP-positive seminiferous tubules in Figure 2G. Scale bar is 100 μm. (G) Offspring obtained by injection of P14BA+E-rPGCLC-derived round spermatids into unfertilized rat oocytes. (H) Expression patterns of *Etv4* in rats (this study) and mice (Shirane et al., 2016). (I) Summary of this study. The gray arrow indicates a mechanism known in mouse model, and the red arrow indicates a potential mechanism uncovered in this study. The three PGC specifiers, enclosed by a gray circle, act together with *Etv4* to activate the germline program. See also Figure S3 and Tables S1 and S2.

To identify the factor(s) regulated by WNT and Activin signals, we conducted a transcriptome analysis to examine differentially expressed genes 24 h after the activation of P14BA, based on the presence or absence of AC supplementation (Figure 4A). To exclude the influence of *Tbxt,* which is not required for P14BA-rPGCLC induction, we used wild-type (WT) and TKO rESC lines with a P14BA-inducible system. *Tbxt* was upregulated upon adding AC in WT but not TKO cells, at 24 h, indicating that *Tbxt* is a downstream target of AC, similar to its role in other mammals (Gadue et al., 2006; Kobayashi et al., 2017; Loh et al., 2014). Among the 26 genes upregulated both in WT and TKO (Log2FC > 2, *p* < 0.05, Figure 4B), we focused on five TFs (*Etv4*, *Foxi3*, *Hnf1b*, *Nkx2.1*, and *Pitx2*) and investigated whether their simultaneous activation, along with P14BA, could induce rPGCLCs in the absence of AC. Among the five candidate genes, we found that *Etv4* and *Hnf1b* could induce N3T-positive cells (Figures 4C and 4D). We further tested whether together, *Etv4* and *Hnf1b* more efficiently induced rPGCLCs, but no synergistic effect was observed (Figure S3E). Since the addition of *Hnf1b* had a modest effect in rPGCLC induction compared to *Etv4*, we focused further on the role of *Etv4*. Use of rN3TAG#3 cell line with P14BA and Etv4 transgenes shows consistent results (Figures S3C and S3D). Interestingly, *Etv4,* but not *Hnf1b,* is upregulated in T-rPGCLCs. We also confirmed that *Etv4* is modestly but rapidly (∼16 h) upregulated after activation of exogenous *Tbxt* (Figure S3F), suggesting that *Tbxt* likely induces *Blimp1, Prdm14,* and *Etv4*, which then work together to promote rPGCLCs induction, even in the absence of both BMP4 and AC. *Etv4* is a known downstream target of glial cell line-derived neurotrophic factor (GDNF) signaling in kidney development (Lu et al., 2009) and fibroblast growth factor (FGF) signaling in limb development (Mao et al., 2009). It plays a critical role in FGF-extracellular signal regulated kinase (ERK) signaling in pluripotent cells (Akagi et al., 2015; Simon et al., 2024; Yang et al., 2024). However, during P14BA-rPGCLC induction, replacing AC with FGF2 fails to induce PGCLCs (Figure S3G), suggesting a context-dependent relationship between external signals and transcriptional regulators. Finally, as a proof of concept, we confirmed that P14BA plus *Etv4*-induced rPGCLCs (P14BA + E-rPGCLCs) can reconstitute spermatogenesis (Figures 4E and 4F; Table S1), resulting in the birth of viable offspring (Figure 4G; Table S2). This result suggests that *Etv4* acts as a downstream target of AC in driving the induction of fully functional rPGCLCs (Figure 4I).

During BMP-rPGCLC induction, we observed that *Etv4* is temporally downregulated in rEpiLCs but re-activated in rPGCLCs (Figure 4H). Interestingly, in mice, *Etv4* expression is modestly upregulated in mEpiLCs compared with mESCs, and is maintained after BMP stimulation (Figure 4H). This may explain why P14BA is sufficient to induce PGCLCs in mice but not in rats. Among the erythroblast transformation specific (ETS) translocation variant (ETV) family members, *Etv4* is a member of the PEA3 subfamily of ETS transcription factors along with *Etv1* and *Etv5. Etv5* is highly expressed in PSCs, and is considered functionally redundant to *Etv4* (Akagi et al., 2015; Kalkan et al., 2019). During the stepwise induction of rPGCLC from rESCs, both *Etv5* and *Etv1* are consistently expressed at similar levels throughout the transition (Figure S3H). Since *Etv4* is upregulated in PGCLCs compared to EpiLCs (Figure 4H), our study suggests that *Etv4* may play a specific role in germline specification. In conclusion, we identified that *Etv4* cooperates with the three PGC specifiers to induce PGC fate in rats, a mechanism that may have been overlooked in mice but was successfully uncovered using our *in vitro* rat system.

## Discussion

In this study, we demonstrate the induction of PGCLCs from EpiLCs through the overexpression of transcriptional regulator(s) in rats, in the absence of BMP4. We found that T-, P14BA-, and P14BA + E-induced rPGCLCs are functionally equivalent to BMP4-induced rPGCLCs in their efficiency to reconstitute spermatogenesis following transplantation (Oikawa et al., 2022). Although a previous report demonstrated that *Tbxt* could induce PGC fate in mice, the subsequent developmental potential of T-induced mPGCLCs after specification has remained unclear (Aramaki et al., 2013). Our data using the rat model clearly demonstrates that T-induced rPGCLCs can normally contribute to gametogenesis, resulting in the birth of viable offspring via ROSI. It should be noted, however, that T-rPGCLCs include rPGCLCs induced by mesoderm-derived BMP. In mice, lower levels of *Tbxt* promote PGC fate, whereas higher levels promote mesodermal fate (Aramaki et al., 2021). Thus, optimal *Tbxt* dosage may be critical for direct induction of rPGCLCs.

Overexpression of transcription factor(s) in bulk selected populations can lead to ambiguous boundaries between the negative and positive populations, likely due to variation in transgene expression. This variability could be due to differences in transgene introduction and subsequent antibiotic selection, resulting in heterogeneous expression levels. To achieve efficient rPGCLC induction, it is crucial to optimize the dosage of each transcription factor. Nevertheless upon T-, P14BA-, and P14BA + E-rPGCLC transplantation into seminiferous tubules, Dox or Dex activation of exogenous transgenes was no longer required. Therefore, once the transcriptional regulatory network to specify PGC fate is established by the exogenous transgenes, subsequent PGC development and gametogenesis proceed independently of the transgenes, cell-autonomously.

PGCLC induction with the three PGC specifiers, *Prdm14*, *Blimp1*, *and Ap2γ*, requires additional Activin and WNT signals in rats. In mice, mEpiLCs are induced as an adherent monolayer, and after dissociation, formation of aggregates activates *Tbxt* (Okashita et al., 2016), likely via the canonical WNT signal pathway. Indeed, *Wnt3* is modestly upregulated in EpiLCs in mice (Hayashi et al., 2011; Nakaki et al., 2013). Since the three PGC specifiers are exogenously activated by adding Dox after aggregate formation in mice, they may function on a state biased toward PS fate. In rats, however, rPGCLCs are induced by simply transferring rEpiLC aggregates to rPGCLC medium without dissociation (Oikawa et al., 2022), this approach may not lead to the induction of PS fate. While BMP4 can induce rPGCLCs, likely through the activation of WNT3 in the epiblast (Aramaki et al., 2013; Ben-Haim et al., 2006), for P14BA-mediated rPGCLC induction, the exogenous activation of WNT and Activin/Nodal signals may be crucial for the appropriate response to P14BA for the establishment of PGC fate in the absence of BMP signal.

From our candidate screening, we identified *Etv4* as a downstream target of Activin and WNT signals. A recent report showed that the expression of *ETV4* varies depending on the size of human PSC colonies and its expression connects cell crowding with lineage specification (Yang et al., 2024). ETV4 positive cells located at the colony edges exhibit mesendodermal fate, while ETV4 negative areas in the colony center are associated with neuroectodermal fate (Yang et al., 2024). Since human PSCs gain competency for PGC fate during their progression toward mesendodermal fate (Kobayashi et al., 2017; Vijayakumar et al., 2023), *ETV4* may play a conserved role in PGC competency across species. In mice, *Etv4* KO animals are sterile due to abnormal spermiogenesis, but not due to defects in PGC specification (Laing et al., 2000). Another study demonstrated that self-renewing formative mPSCs can generate PGCLCs in the absence of both *Etv4* and *Etv5*, suggesting that other factor(s) may compensate for the function of *Etv4* during PGC specification (Kinoshita et al., 2021). Since the efficiency of rPGCLC induction by P14BA + E is still lower than that of P14BA with AC, additional factor(s) may also be involved in regulating PGC specification.

The rat as a model can be leveraged for its similarities and differences to the mouse model, making it valuable for testing whether findings in mice are conserved across species or specific to mice. For instance, in our study, validating mouse results in the rat model led to the identification of a novel transcriptional regulator, *Etv4*, which cooperates with the three previously identified PGC specifiers to induce PGC fate. Similar to PSC culture and PGCLC induction, capturing and reconstituting rat embryonic development often requires more stringent conditions with less redundancy compared to mice. Thus, further investigation using the rat model, to review and validate findings in mice, as demonstrated in our study will provide deeper mechanistic insights into early embryo and germline development. This could ultimately pave the way for the development of widely applicable *in vitro* gametogenesis techniques in the future.

## Methods

### Culture and genetic manipulation of rESCs

Male rESC lines harboring Nanos3-tdTomato reporter and Acrosin-EGFP reporter (rN3TAG#2, #3) was derived previously (Oikawa et al., 2022). The rESCs were routinely cultured in rESC medium: N2B27 medium containing 2i (PD0325901, 1 μM, Axon, Groeningen, The Netherlands; CHIR99021, 3 μM, Axon) and rat LIF (1,000 U/mL, Merck Millipore, MA). All the components of N2B27 were purchased from Thermo Fisher Scientific according to published protocol (Hirabayashi et al., 2019; Oikawa et al., 2024). The rESCs were passaged by 0.25% Trypsin-EDTA (Thermo Fisher Scientific) every 2–3 days according to our published protocol (Hirabayashi et al., 2019; Oikawa et al., 2024). For introducing exogenous transgenes, PiggyBac vectors containing Dox-inducible or Dex-inducible systems previously developed (Kobayashi et al., 2017) were used accordingly (Figures 1A and S2A). All rat cDNAs were amplified by PCR using PrimeSTAR MAX DNA polymerase (Takara Bio, Shiga, Japan) or KOD One DNA polymerase (Toyobo, Osaka, Japan) according to the manufacturer’s protocol. For generating Tbxt KO rESCs, gRNA sequence described in Figure 3A is subcloned into eSpCas9(1.1) (Addgene #71814) CRISPR/Cas9 vector. PiggyBac vectors with PBase plasmid or CRISPR/Cas9 vector were transfected into rESCs by reverse transfection method. For PiggyBAC vectors, the transfected rESCs were seeded on a Neomycin and Puromycin resistance feeder generated in-house (Ohtsuka et al., 2015), and 48 h later, 0.8–1.0 μg/mL puromycin (Sigma-Aldrich, MO) and/or 200 μg/mL G418 (Sigma-Aldrich) were added to the culture medium for selection.

### Induction of rPGCLCs

The protocol is essentially the same as our published one (Oikawa et al., 2022, 2024). rESCs were dissociated using 0.25% Trypsin-EDTA and harvested in 96-well Nunclon Sphera-Treated U-shaped microplate (Thermo Fisher Scientific) containing 100 μL of rEpiLC medium at 4 × 10^3^ cells per well. rEpiLC medium composed of N2B27 medium containing Activin A (20 ng/mL, Peprotech), bFGF (12 ng/mL; Peprotech), and 1% KSR (Thermo Fisher Scientific) was prepared freshly on the day of use. On day 1 of rEpiLC induction, each well was topped up with 100 μL of fresh medium. On day 2, half volume of the medium was replaced with fresh medium. On day 2–3 after induction, the rEpiLCs were washed in PBS containing 3% fetal bovine serum (FBS) or N2B27 containing 5% KSR using a glass capillary. Then, rEpiLCs were transferred into a well of 96-well Nunclon Sphera-Treated U-shaped microplate containing 100 μL of freshly made rPGCLC medium composed of N2B27 medium containing BMP4 (500 ng/mL; Peprotech), rat LIF (1000 U/mL; Merck Millipore), mSCF (100 ng/mL; R&D systems), mEGF (50 ng/mL; R&D systems), and 5% KSR. For induction of exogenous transgenes, 100 μM Dex (Sigma), or 1 μg/mL Dox (Sigma) were added to the medium instead of BMP4 or other cytokines. To test signaling inhibitors, TGF-β Type I Receptor inhibitor SB-431542 (10 μM; Selleck Chemicals, TX), WNT inhibitors IWP-2 (1 μM; Tocris Bioscience, Bristol, UK) and XAV939 (5 μM; Cayman Chemical; MI), or LDN-193189 (0.5 μM; Tocris Bioscience) were used, respectively. At day 3 after rPGCLC induction, rPGCLCs were subjected to the downstream analysis. The morphology and fluorescent of rPGCLCs were observed using BZ-X810 (Keyence, Osaka, Japan).

## Resource availability

### Lead contact

Further information and requests for resources and reagents should be directed to the lead contact, Toshihiro Kobayashi (tkoba@nips.ac.jp).

### Materials availability

Unique reagents generated in this study are available from the lead contact with a materials transfer agreement.

### Data and code availability

• This paper analyzes existing, publicly available data. These accession numbers for the datasets are listed in the key resources table. RNA-seq data had been deposited in the Sequence Read Archive (SRA) under BioProject ID: PRJNA1199573.
• Any additional information required to reanalyze the data reported in this paper is available from the lead contact upon request.

## Acknowledgments

We thank members of the Kobayashi lab and Hirabayashi lab, particularly Keiko Yamauchi and Fumika Yoshida, for technical assistance, and Minako Ohnishi for secretarial support. We also thank Dr. Roopsha Sengupta for editing and providing critical input to the manuscript. Flow cytometry was performed in the IMSUT FACS Core laboratory. This work was supported by JSPS 10.13039/501100001691KAKENHI 18H02367, 20H03167, 23K20043, 24K01944, and 25H01349 to T.K.; 18H05544 to T.K. and K.K.; 19K23711 to M.O.; 21H02382 to H. Kobayashi; 21J21849 to K.I.; 21J21849, 10.13039/100009619AMED JP18bm0704022, and JP22bm1123008 to T.K.; JST JPMJFR233J to T.K.; Cooperative Study Program 24NIPS310 of NIPS to T.K.; and grants from 10.13039/100008608The Sumitomo Foundation 210348 to T.K., 10.13039/501100004051Kato Memorial Bioscience Foundation to T.K., and 10.13039/501100005927Daiichi Sankyo Foundation of Life Science to T.K.

## Author contributions

Conceptualization: M.O. and T.K.; methodology: M.O., H. Kojima, K.I., H. Kobayashi, K.N., M.S., T. Yamamoto, M.H., and T.K.; formal analysis: M.O. and T.K.; investigation: M.O. and T.K.; visualization: M.O. and T.K.; writing: M.O. and T.K.; supervision: T. Yamaguchi, K.K., and T.K.; funding acquisition: T.K.

## Declaration of interests

The authors declare no competing interests.

## Supplemental information

Document S1. Figures S1–S3, Tables S1–S4, and supplemental methods

Document S2. Article plus supplemental information
