# Supplementary material for: Transcription factor-mediated germ cell induction in rats reveals ETV4 cooperates with germline specifiers
Source: Stem Cell Reports. 2025 Aug 12;20(8):102599. doi: 10.1016/j.stemcr.2025.102599 (PMC12365847; doi:10.1016/j.stemcr.2025.102599)
Supplement: Document S1. Figures S1–S3, Tables S1–S4, and supplemental methods [file mmc1.pdf]

Stem Cell Reports, Volume *20*

Supplemental Information

Transcription factor-mediated germ cell induction in rats reveals ETV4 cooperates with germline specifiers

Mami Oikawa, Hiroki Kojima, Hisato Kobayashi, Kenyu Iwatsuki, Hijiri Saito, Makoto Sanbo, Kazumi Nishioka, Tomoyuki Yamaguchi, Takuya Yamamoto, Kazuki Kurimoto, Masumi Hirabayashi, and Toshihiro Kobayashi

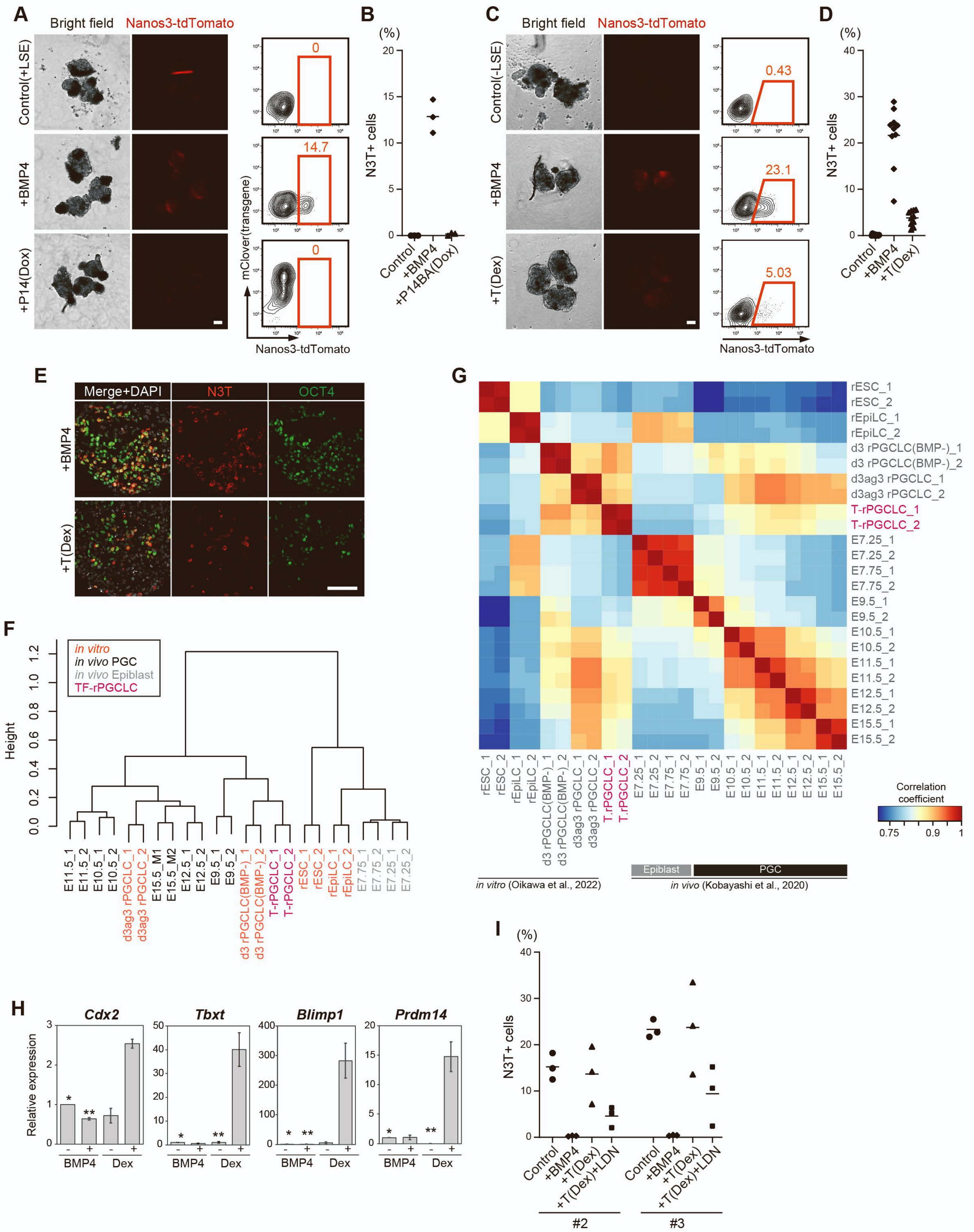

Oikawa et al., Figure S1

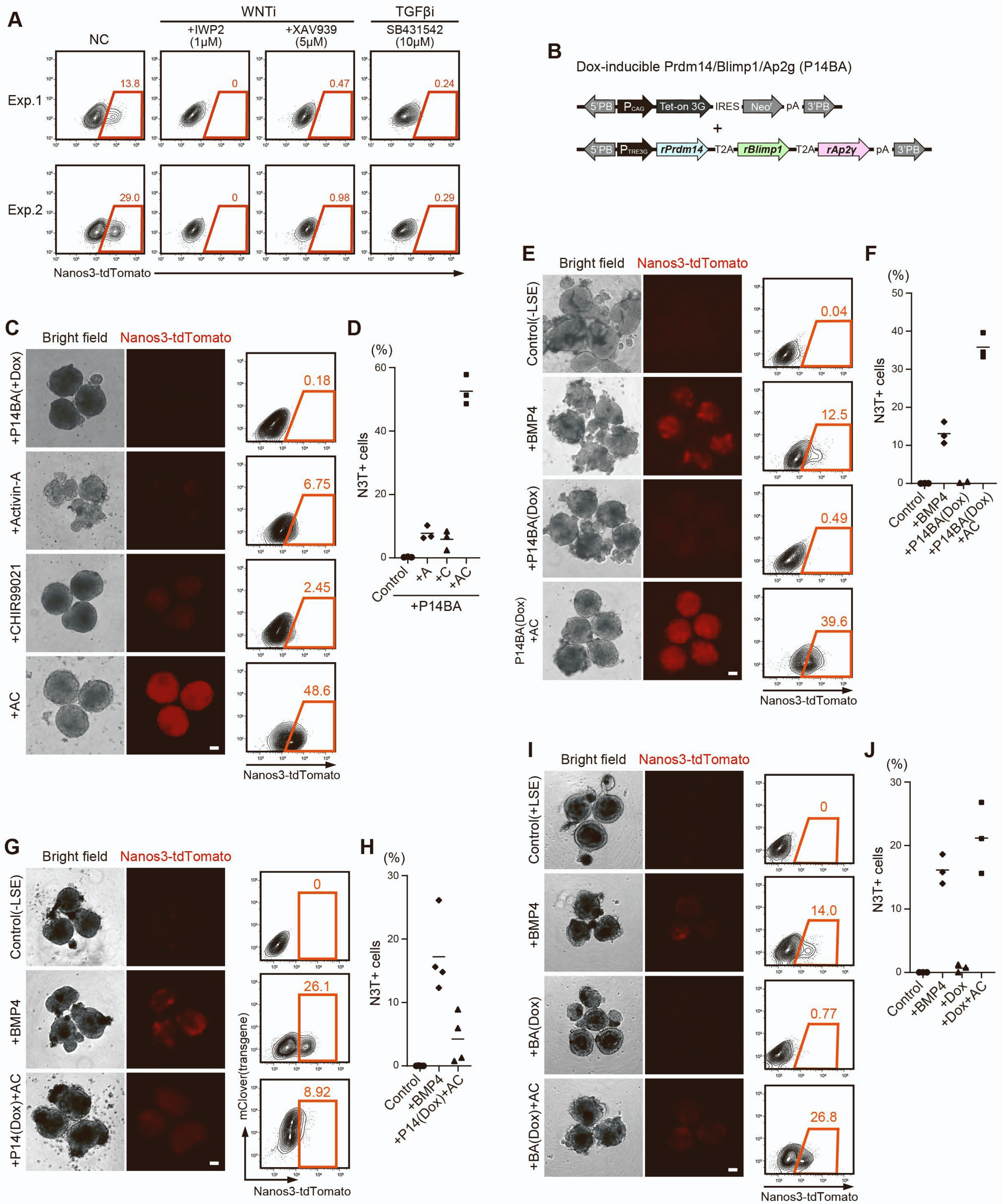

Oikawa et al., Figure S2

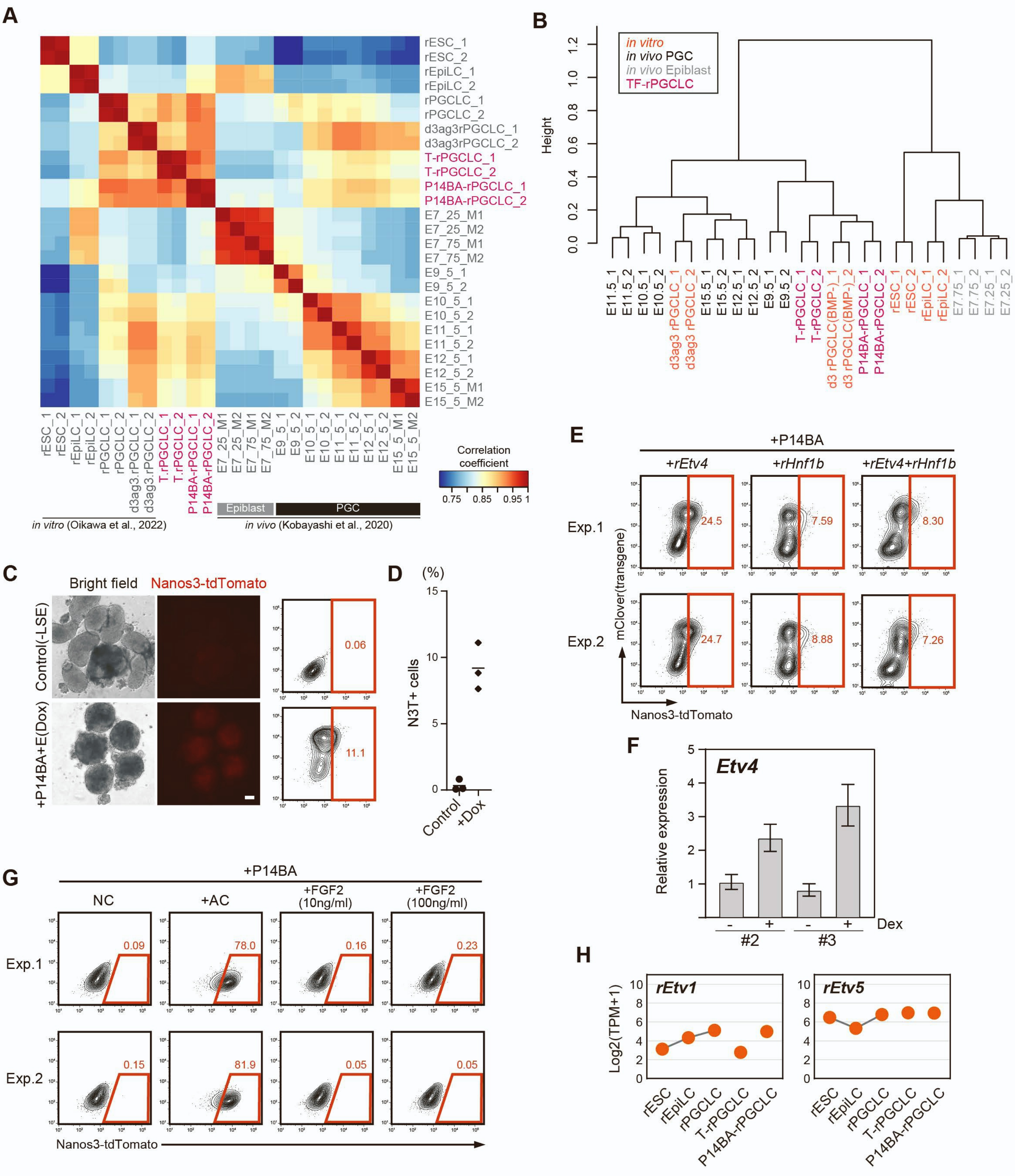

Oikawa et al., Figure S3

SUPPLEMENTARY FIGURE TITLES AND LEDENDS

Figure S1. Characterization of T-rPGCLC, related to Figure 1

(A) Images and FACS patterns of day 3 rPGCLCs induced by only LSE, LSE plus BMP4, and LSE plus P14 by adding Dox. Scale bar is 100 µm.

(B) Dot plot showing percentage of Nanos3-tdTomato (N3T) positive cells in **Figure S1A** (n = 3 biologically independent experiments).

(C) Images and FACS patterns of day 3 rPGCLCs induced by cytokine-free condition, BMP4, and T by adding Dex. Scale bar is 100 µm.

(D) Dot plot showing percentage of Nanos3-tdTomato (N3T) positive cells in **Figure S1C** (n = 10 biologically independent experiments).

(E) IF images of day 3 rPGCLCs induced by BMP4 or T. Scale bar is 100 µm.

(F) Hierarchical clustering among T-rPGCLCs and published in vitro/in vivo samples as indicated.

(G) Heatmap of the correlation coefficients among T-rPGCLCs and published in vitro/in vivo samples as indicated.

(H) RT-qPCR analysis of cells at 16 h after induction of rPGCLCs by BMP4 or exogenous *Tbxt* by Dex. Relative expression levels to BMP4 minus controls are shown. Values are normalized in reference to *rActb*. Bars represent averages of n = 3 biologically independent experiments. One or two asterisks indicate Ct values detected in only one or two samples due to low expression levels.

(I) Dot plot showing percentage of Nanos3-tdTomato (N3T) positive cells in BMP- and T-rPGCLCs with or without BMP inhibitor (n = 3 biologically independent experiments, 2 independent cell lines).

Figure S2. Characterization of P14BA-rPGCLC, related to Figure 2

(A) FACS patterns of day 3 rPGCLCs induced by BMP4 plus LSE with or without Activin receptor inhibitor, SB431542 (10uM), or WNT inhibitors, XAV939 (5uM) and/or IWP-2 (1uM) (n = 2 biologically independent experiments).

(B) A polycistronic gene-inducible system in this study; Dox-inducible *rPrdm14-T2A-rBlimp1-T2A-rAp2*γ.

(C) Images and FACS patterns of day 3 rPGCLCs induced by P14BA with or without Activin A and CHIR99021 as indicated. Scale bar is 100 µm.

(D) Dot plot showing percentage of Nanos3-tdTomato (N3T) positive cells in **Figure S2B** (n = 3 biologically independent experiments).

(E) Images and FACS patterns of day 3 rPGCLCs from rN3TAG#3 induced by P14BA. Scale bar is 100 µm.

(F) Dot plot showing percentage of Nanos3-tdTomato (N3T) positive cells in **Figure S2E** (n = 2-3 biologically independent experiments).

(G) Images and FACS patterns of day 3 rPGCLCs induced by cytokine free condition, BMP4, and P14 by adding Dox with AC. Scale bar is 100 µm.

(H) Dot plot showing percentage of Nanos3-tdTomato (N3T) positive cells in **Figure S2D** (n = 4 biologically independent experiments).

(I) Images and FACS patterns of day 3 rPGCLCs induced by only LSE, BMP4 plus LSE, and BA by adding Dox with or without AC. Scale bar is 100 μm.

(J) Dot plot showing percentage of Nanos3-tdTomato (N3T) positive cells in **Figure S2F** (n = 3 biologically independent experiments).

Figure S3. Effects of signals and transgenes for rPGCLC induction, related to Figure 2 and 4

(A) Hierarchical clustering among P14BA-rPGCLCs and published in vitro/in vivo samples as indicated.

(B) Heatmap of the correlation coefficients among P14BA-rPGCLCs and published in vitro/in vivo samples as indicated.

(C) Images and FACS patterns of day 3 rPGCLCs from rN3TAG#3 induced by P14BA+E. Scale bar is 100 μm.

(D) Dot plot showing percentage of Nanos3-tdTomato (N3T) positive cells in **Figure S3C** (n = 3 biologically independent experiments).

(E) FACS patterns of day 3 rPGCLCs induced by P14BA together with *rEtv4* and/or *Hnf1b*. (n = 2 biologically independent experiments)

(F) RT-qPCR analysis of cells at 16 h after induction of rPGCLCs by exogenous *Tbxt* by Dex. Relative expression levels to Dex minus control in rN3TAG#2 are shown. Values are normalized in reference to *rActb*. Bars represent averages of n = 2 biologically independent experiments.

(G) FACS patterns of day 3 rPGCLCs induced by P14BA with AC or FGF2.

(H) Expression patterns of *Etv1,* and *Etv5* in rats.

TF-rPGCLCs	No. of testes transplanted	No. of testes with successful transfer	No. of Testes with EGFP positive tubules (%)	No. of EGFP positive seminiferous tubules
T-rPGCLC	6	5/6 (83)	5/5 (100)	3, 4, 2, 2, 5
P14BA-rPGCLC	8	7/8 (88)	7/7 (100)	>5, 3, >5, 3, >5, >5, 2
P14BA+E-rPGCLC	5	4/5 (80)	3/4 (75)	>5, 5, 1

Oikawa et al., Table S1

Transplanted TF-rPGCLC	Injected cell type	No. of oocyes survived after injection	No. of oocytes forming pronulei (%)*	No. of cleaved embryos (%)*	No. of embryos transferred	No. of full-term pups (%)**
T-rPGCLC	Spermatid	69	46 (67)	8 (12)	66	3 (5)
P14BA-rPGCLC	Spermatid	94	65 (69)	9 (10)	88	4 (5)
P14BA+E-rPGCLC	Spermatid	125	79 (63)	4 (3)	114	5 (4)

* Percentages for forming pronuclei and first cleaved embryos were calculated from the number of oocytes survived after injection.

** Percentages for full-term pups were calculated from the number of embryos transferred.

Oikawa et al., Table S2

Targeted genes	Primer ID	Sequence
rCdx2	MO231_rCdx2_F	ACAGAAAGCTGGATTGACCGA
	MO232_rCdx2_R	TCACACGATGGTCCCTGAAC
rTbxt	MO186_rTbxt_F	ATGTCCTCCCTTGTCGCTTTAG
	MO187_rTbxt_R	CGGTTCAGTTACAATCCGCTG
rBlimp1	MO095_rPrdm1_For	AGGATGTGGACTGGGTGGAC
	MO096_rPrdm1_Rev	CTTCACGGAACCGGAGTTACA
rPrdm14	MO043_rPrdm14_For	TCTCGGATGTGGGAAATTTTTGA
	MO044_rPrdm14_Rev	GGGGAATCGAGCACAGTTGA
rActb	MO061_rActb_For	CCCGCGAGTACAACCTTCTT
	MO062_rActb_Rev	CGACGAGCGCAGCGATA

Oikawa et al., Table S3

Antibody/Lectin	Company	Cat No.	RRID	Dilution
anti-GFP	Abcam	ab13970	AB_300798	1:500
anti-TFAP2C	SantaCruz	sc-8977	AB_2286995	1:250
anti-OCT3/4	SantaCruz	sc-5279	AB_628051	1:250
anti-DsRed	Takara Bio Inc.	632496	AB_10013483	1:500
anti-mCherry	EnCor	CPCA-mCherry	AB_2572308	1:250
anti-SOX9	Abcam	ab185966	AB_2728660	1:250
anti-PNA	Vector	RL-1072	AB_2336642	1:300

Oikawa et al., Table S4

SUPPLEMENTARY TABLE TITLES

Table S1 Efficiency of spermatogenesis after transplantation of TF-rPGCLCs into seminiferous tubules of neonatal *Prdm14* KO testis, related to Figure 1, 2, and 4

Table S2 Development of embryos fertilized with spermatid derived from TF-rPGCLCs, related to Figure 1, 2, and 4

Table S3 Primer sequences for RT-qPCR, related to Figure S1 and S3

Table S4 Antibodies used for IF, related to Figure 1, 2, and Figure S1

SUPPLEMENTAL METHODS

Animals

Crlj:WI (RGD ID: 2312504) rats were purchased from Charles River Laboratories Japan, Inc. (Kanagawa, Japan). Slc:SD (RGD ID: 12910483) rats were purchased from SLC Japan (Shizuoka, Japan). All experiments were performed in accordance with the animal care and use committee guidelines of the National Institutes of Natural Sciences and University of Tokyo.

Transplantation of the rPGCLCs into seminiferous tubules

About 60-120 of rPGCLC aggregates were prepared for one experiment. Aggregates at day 3 after induction of rPGCLC were dissociated with 500 µl of 0.25% Trypsin-EDTA at 37 ℃ for 5 min. The reaction was terminated by adding 2 ml of DMEM containing 10% FBS, 1% Glutamax and 1% penicillin/streptomycin. FACS-sorted N3T(+) d3 rPGCLCs were collected and suspended into rPGCLC medium at the concentration of $1x10^4$ cells per 2 µl. 0.5 µl of Trypan blue was added (total ~2.5 µl) to the cell suspensions to confirm the successful injection into the efferent duct, visually. For the recipient, genotyped day 5-7 *Prdm14*$^{H2BVenus/mut}$ (KO) neonatal rats were anesthetized with Isoflurane. A glass capillary filled with 2.5 µl of rPGCLC suspensions was carefully punctured into efferent duct of the testis and injected the cell suspensions using FemtoJet injection system (Eppendorf, Hamburg, Germany). After injection, neonatal rats that underwent surgery were kept on the 37 ℃ warming plate for at least 30 min and returned to the mother rats.

Round spermatid injection

Round spermatids were collected from EGFP positive seminiferous tubules. Cells were dissociated and suspended in GL-PBS (Dulbecco's PBS supplemented with 5.6 mM glucose, 5.4 mM sodium lactate and 0.01% polyvinylpyrrolidone) at 4˚C. Round spermatids were selected based on their appearance or collected with the SH800 cell sorter (SONY, Tokyo, Japan) according to a published protocol [35]. Metaphase II stage oocytes were collected in HEPES-R1ECM medium from superovulated Slc:SD rats and denuded the cumulus with 0.1% hyaluronidase. Oocytes were activated with 5 mM ionomycin for 5 min, and incubated for 40 min in mR1ECM medium until injection. ROSI embryos were treated for 4 h with 5 µg/mL cycloheximide (Sigma-Aldrich) in mR1ECM. Embryos were washed with mR1ECM and cultured in a humidified atmosphere of 5% CO_2 at 37 ℃. On the next day, both 2-cell and 2PN formed embryos were transferred into an oviduct of 0.5 dpc pseudo-pregnant Crlj:WI rats. On 21.5 dpc, the recipient females were subjected to a Caesarean section.

Fluorescence-activated cell sorting (FACS)

Cells were dissociated with 0.25% Trypsin-EDTA at 37 ℃ for 5 min. The reaction was terminated by adding 10X volumes of DMEM containing 10% FBS, 1% Glutamax and 1% penicillin-streptomycin. Non-dissociated cells were removed by 70 µm cell strainer then resuspended into FACS buffer (3% FBS in PBS or 0.1% BSA in PBS). The cells were analyzed or sorted using the flow cytometers (SH800 and MA900; SONY or Aria III; BD Biosciences). FACS data were re-

analyzed by Flowjo software (BD Biosciences)

Quantitative reverse transcription PCR

Total RNA was extracted using PicoPure® RNA Isolation Kit and cDNA was synthesized using QuantiTect Reverse Transcription Kit (QIAGEN, Venlo, The Netherland) according to the manufacturer's protocols. RT-qPCR were performed and analyzed as described previously and the primers sequences used in the paper are listed in **Table S3**.

Immunofluorescence analysis

Samples were fixed with 4% paraformaldehyde for 10-30 min at RT or from 4 h to overnight at 4 ℃ depending on the experiments. For making cryosections, samples were treated with a gradient of 10%, 20%, and 30% sucrose and then embedded in OCT compound (Sakura Finetek, Tokyo, Japan). Samples were cut into 7 µm-thick cryosections using Cryostat (Leica Biosystems, Wetzlar, Germany). After air drying, the sections were washed with PBS, PBS with 0.1% Triton X and then incubated with blocking buffer: 5% normal donkey serum (Sigma-Aldrich), 1% BSA, 0.1% Triton X in PBS. Sections were incubated with primary antibodies for 1-2 h at RT or overnight at 4 ℃. After washing with PBS with 0.1% Triton X, the sections were incubated with fluorescent-conjugated secondary antibodies with DAPI (Dojindo) for 1 h at RT or overnight at 4 ℃. After washing with 0.1% Triton X in PBS, samples were mounted with mounting medium. At least 3 sections were analyzed for each condition. Antibodies used are listed on **Table S4**. Specimens were observed and analyzed using FV3000

(Olympus; Olympus, Tokyo, Japan).

Preparation of RNA-sequencing libraries

Total RNA was extracted using the PicoPure RNA Isolation Kit following the manufacturer’s protocol. cDNA library was constructed using SMART-Seq v4 Ultra Low Input RNA Kit (Takara Bio) or SMART-Seq HT PLUS Kit (Takara Bio) and following the manufacturer's recommendations. For Illumina sequencing, cDNA was synthesized from 1 ng of total RNA with 10-15 cycles of PCR amplification, subsequently, 5 ng of cDNA was used for addition of Illumina’s adaptors with 13-15 cycles of PCR amplification. The quality and quantity of RNA-seq libraries were evaluated by qPCR using KAPA Library Quantification Kit (Kapa Biosystems). All libraries were pooled and applied to single-end 86 bp sequencing on NextSeq 500 system (Illumina, San Diego, CA) using High Output Kit v2.5. Basecalls were performed using NextSeq 500/550 RTA software (v2.11.3). FASTQ files were generated using bcl2fastq (v2.17.1.14). Two technical replicates of all samples were used for the analysis. RNA-seq data had been deposited in the Sequence Read Archive (SRA) under BioProject ID: PRJNA1199573.

Bioinformatics analysis

For processing the RNA-seq data, we used RaNA-seq program (https://ranaseq.eu/index.php) to calculate TPM values. For further analysis, rat genes showing maximum log2 (TPM+1) values >4 in at least one replicate were selected (9133 genes). Hierarchical clustering was performed based on Ward’s

method using the ‘htclust’ function of R package. The PCA was performed using the ‘tidyverse’ function of the R package.

Declaration of generative AI and AI-assisted technologies in the writing process

During the preparation of this work the author(s) used ChatGPT in order to improve refining language and grammar. After using this tool, the authors reviewed and edited the content as needed and take full responsibility for the content of the publication.
