# Supplementary material for: Transcription factor-mediated germ cell induction in rats reveals ETV4 cooperates with germline specifiers
Source: Stem Cell Reports. 2025 Aug 12;20(8):102599. doi: 10.1016/j.stemcr.2025.102599 (PMC12365847; doi:10.1016/j.stemcr.2025.102599)
Supplement: Document S2. Article plus supplemental information [file mmc2.pdf]

Stem Cell Reports
Report
OPEN ACCESS

Transcription factor-mediated germ cell induction in rats reveals ETV4 cooperates with germline specifiers

Mami Oikawa,[1,2,10] Hiroki Kojima,[1,2,5,10] Hisato Kobayashi,[3,4] Kenyu Iwatsuki,[1,6] Hijiri Saito,[1] Makoto Sanbo,[5] Kazumi Nishioka,[5] Tomoyuki Yamaguchi,[2] Takuya Yamamoto,[6,7,8] Kazuki Kurimoto,[3] Masumi Hirabayashi,[5] and Toshihiro Kobayashi[1,5,9,11,*]

[1]Division of Mammalian Embryology, Center for Stem Cell Biology and Regenerative Medicine, The Institute of Medical Science, The University of Tokyo, Minato-ku, Tokyo 108-8639, Japan
[2]Laboratory of Regenerative Medicine, Tokyo University of Pharmacy and Life Science, Hachioji, Tokyo 192-0392, Japan
[3]Department of Embryology, Nara Medical University, Kashihara, Nara 634-8521, Japan
[4]Department of Medical Genome Science, Dokkyo Medical University, Mibu, Tochigi 321-0293, Japan
[5]Division of Mammalian Embryogenesis, Department of Homeostatic Regulation, National Institute for Physiological Sciences, Okazaki, Aichi 444-8787, Japan
[6]Center for iPS Cell Research and Application, Kyoto University, Sakyo-ku, Kyoto 606-8507, Japan
[7]Institute for the Advanced Study of Human Biology, Kyoto University, Sakyo-ku, Kyoto 606-8501, Japan
[8]Medical-risk Avoidance based on iPS Cells Team, RIKEN Center for Advanced Intelligence Project, Sakyo-ku, Kyoto 606-8507, Japan
[9]The Graduate University of Advanced Studies, Okazaki, Aichi 444-8787, Japan
[10]These authors contributed equally
[11]Lead contact
*Correspondence: tkoba@nips.ac.jp
https://doi.org/10.1016/j.stemcr.2025.102599

SUMMARY

The specification of primordial germ cells (PGCs) marks a crucial branchpoint in early embryonic development. Studying the molecular mechanisms governing this process is crucial for understanding reproduction and evolution. Here, we identify transcription factors essential for PGC specification in rats using an *in vitro* system to induce PGC-like cells (PGCLCs) from pluripotent cells. Overexpression of *Tbxt*, a key mesodermal factor activating the germ cell program in epiblast-like cells, induces functional rat PGCLCs, similar to mice. However, unlike in mice, overexpression of the PGC specifiers (*Prdm14*, *Blimp1*, and *Ap2γ*) alone is not sufficient in rats; additional Activin and WNT signals are necessary for PGCLC induction. Through a candidate screen, we identified the transcription factor *Etv4* acting cooperatively with the three PGC specifiers. Our study provides insight into the mechanism behind germline segregation in mammals and underscores the importance of using the rat model in addition to mice.

INTRODUCTION

The germline, a unique cell lineage capable of transmitting genetic information across generations, is one of the first lineages to segregate from pluripotent cells during early mammalian development (T. Kobayashi and Surani, 2018). Studies on the extrinsic and intrinsic factors that specify primordial germ cells (PGCs), the founder cells for sperm and eggs, offer fundamental insights into cell fate decisions. In mice, the proximal posterior epiblast is directed toward PGC fate through the successive action of extrinsic wingless (WNT) and bone morphogenetic protein (BMP) signals, as demonstrated by knockout (KO) studies and *ex vivo* epiblast cultures (Ohinata et al., 2009; Ying et al., 2001). Understanding the intrinsic transcriptional program has been technically challenging due to the small number of specified PGCs (<40). However, this limitation has been addressed through recent advancements in the development of *in vitro* systems inducing PGC-like cells (PGCLCs) from pluripotent stem cells (PSCs). In this system, the transition from naive mouse PSCs to formative epiblast-like cells (EpiLCs) recapitulates post-implantation epiblast development. Subsequently, the formation of aggregates in the presence of BMP4, a cytokine critical for PGC fate, efficiently induces PGCLCs (Hayashi et al., 2011).

Using this robust *in vitro* system, Nakaki et al., demonstrated that overexpression of three germ cell specifiers, *Prdm14*, *Blimp1* (also known as *Prdm1*), and *Ap2γ* (also known as *Tfap2c*) (Ohinata et al., 2005; Weber et al., 2010; Yamaji et al., 2008), in mouse EpiLCs (mEpiLCs) efficiently induces mouse PGCLCs (mPGCLCs) without the need for external BMP (Nakaki et al., 2013). Among the three PGC specifiers, *Prdm14* plays a central role in inducing the germline program as well as epigenetic reprogramming in mice (Nakaki et al., 2013; Seki, 2018). While, *Tbxt* (*Brachyury, T*), a mesodermal factor, can also direct epiblast toward PGC fate by activating the PGC specifiers (Aramaki et al., 2013). These *in vitro* systems have also been applied to other mammals, including human PSCs to investigate the molecular mechanisms underlying PGC specification (Kobayashi et al., 2017; Kojima et al., 2017; Kojima et al., 2021; Tang et al., 2022).

Recently, we successfully established an *in vitro* system to induce PGCLCs in rats, a widely used experimental rodent animal model alongside mice (Oikawa et al., 2022). We found that, unlike adherent monolayer cultures in mice,

© 2025 The Author(s).
Published by Elsevier Inc. on behalf of International Society for Stem Cell Research.
This is an open access article under the CC BY license (http://creativecommons.org/licenses/by/4.0/).

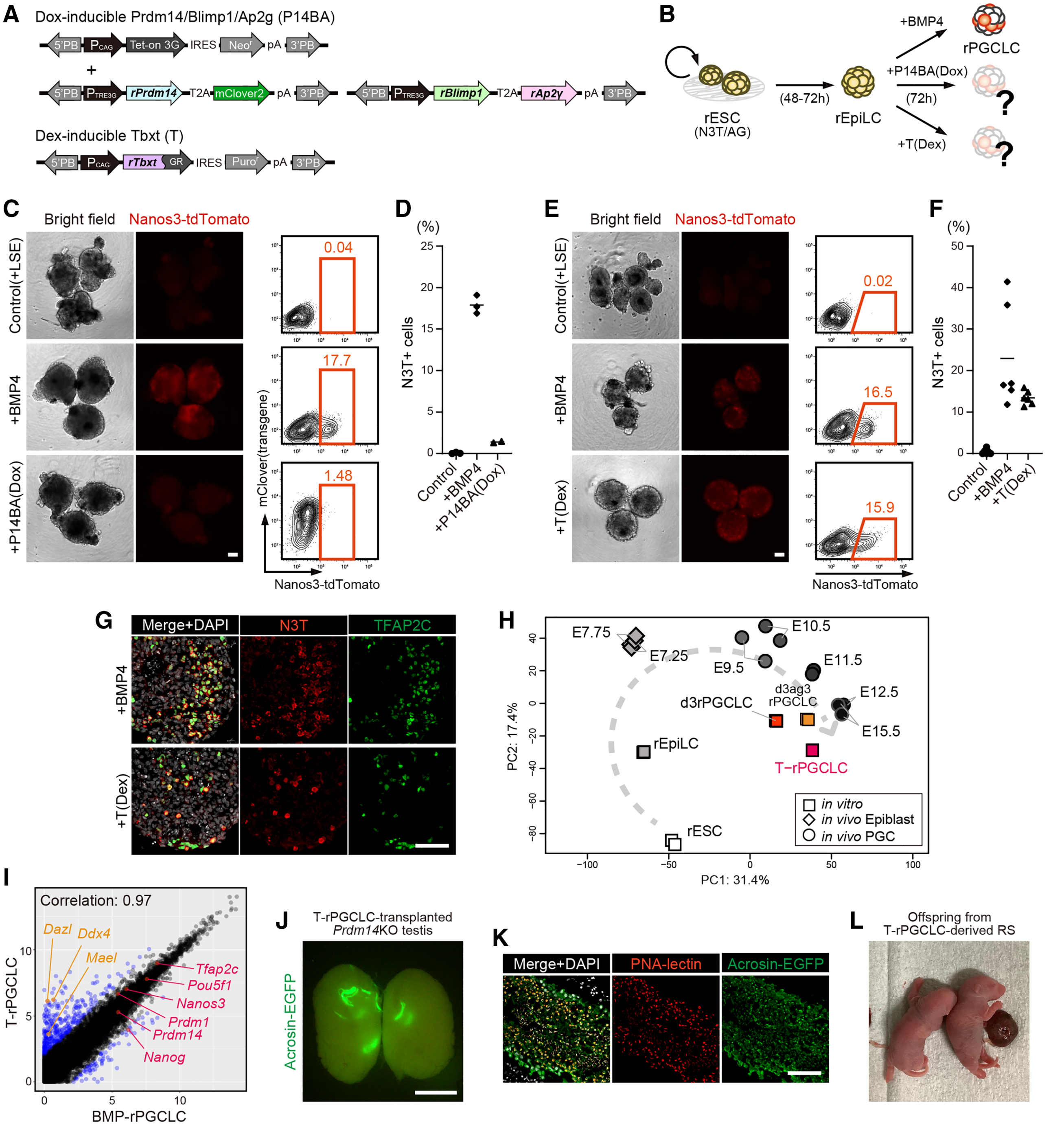

Figure 1. *Tbxt* but not *Prdm14/Blimp1/Ap2γ* can induce functional rPGCLC in the absence of BMP4

(A) Gene-inducible system in this study; Dox-inducible *rPrdm14-T2A-mClover2*, *rBlimp1-T2A-rAp2γ*, and Dex-inducible *rTbxt*.
(B) Experimental design of transcription factor(s)-mediated rPGCLC induction.
(C) Images and FACS patterns of day 3 rPGCLCs induced by only LSE, LSE plus BMP4, and LSE plus P14BA by adding Dox. Scale bar is 100 μm.
(D) Dot plot showing percentage of Nanos3-tdTomato (N3T)-positive cells in Figure 1C (*n* = 2–3 biologically independent experiments).
(E) Images and FACS patterns of day 3 rPGCLCs induced by only LSE, LSE plus BMP4, and LSE plus T by adding Dex. Scale bar is 100 μm.
(F) Dot plot showing percentage of Nanos3-tdTomato (N3T)-positive cells in Figure 1E (*n* = 6 biologically independent experiments).
(G) IF images of day 3 rPGCLCs induced by BMP4 or T. Scale bar is 100 μm.

(legend continued on next page)

rat EpiLCs induced from pluripotent embryonic stem cells (rESCs) require the formation of spherical aggregates for PGCLC induction. Notably, rat PGCLCs (rPGCLCs) are fully functional and able to complete spermatogenesis upon transplantation into seminiferous tubules of germ cell-free *Prdm14* KO rats (Kobayashi et al., 2020, 2021). Furthermore, the rPGCLC-derived spermatids/sperm contribute to the birth of offspring, a result that had only been previously reported for mice.

Utilizing this reliable *in vitro* system and *in vivo* stringent assay, here, we investigate the transcriptional factor(s) (TF (s)) driving PGC fate in rats. We find that TFs important for PGC specification found in mice are well conserved in rats. However, supplementation of Activin and WNT signals is necessary to induce functional PGCLCs by the three PGC specifiers (*Prdm14*, *Blimp1*, *and Ap2γ*). Furthermore, we identify *Etv4*, a potential downstream target gene of these signals, which acts cooperatively to induce PGC fate.

RESULTS

Tbxt induces functional PGCLCs in rats

To verify whether the key TFs directing mouse PGC fate are conserved in rats, we first tested if rEpiLCs are competent to induce rPGCLCs in response to exogenous TF(s) in the absence of BMP4. Based on previous reports from the mouse model (Aramaki et al., 2013; Nakaki et al., 2013), doxycycline (Dox)-inducible *Prdm14-T2A-mClover2* (P14) alone, P14 together with *Blimp1-T2A-Ap2γ* (P1A), or dexamethasone (Dex)-inducible *Tbxt* (T) were introduced into rESCs (Figure 1A). For rESCs, we used two independent double reporter male rESC lines (rN3TAG#2 and #3) harboring both PGC-specific *Nanos3-T2A-tdTomato* (N3T) and spermatogenic cell-specific *Acrosin-EGFP* (AG) (Oikawa et al., 2022). Unless otherwise specified, the representative data shown in the figures were obtained using rN3TAG#2. P14-, P14BA- or T-inducible N3T/AG-rESCs were differentiated into rEpiLC aggregates for 48–72 h; the aggregates were then transferred into N2B27 + 5% knockout serum replacement (KSR) medium containing cytokines (rat leukemia inhibitory factor [LIF], mouse stem cell factor [mSCF], and mouse epidermal growth factor [mEGF], hereafter, LSE) with or without Dox/Dex (Figure 1B). In contrast to the previous data obtained from the mouse model, neither P14 nor P14BA induced N3T-positive rPGCLCs (Figures 1C, 1D, S1A, and S1B). Instead, consistent with mouse data, exogenous T successfully induced N3T expression at nearly similar efficiency and intensity as BMP4-induced rPGCLCs (hereafter, BMP-rPGCLCs) (Figures 1E and 1F). We also found that T was sufficient to induce N3T-positive rPGCLCs even in the absence of LSE cytokines, although with a lower efficiency (Figures S1C and S1D). Immunofluorescence (IF) analysis revealed that T-induced N3T-positive cells express the pluripotency and germ cell markers, TFAP2C and OCT4 (Figures 1G and S1E). We next analyzed the gene expression profiles of T-induced rPGCLCs (hereafter denoted as T-rPGCLC) by RNA sequencing (RNA-seq) and compared the transcriptomic data with *in vivo* rPGC and *in vitro* rPGCLC from our previous datasets (Kobayashi et al., 2020; Oikawa et al., 2022). Hierarchical clustering, correlation heatmap, and principal-component analysis (PCA) revealed that the transcriptome of T-rPGCLCs is highly similar to that of BMP-rPGCLCs, closely corresponding to embryonic day (E)9.5–12.5 *in vivo* rat PGCs (Figures 1H, 1I, S1F, and S1G). Both T-rPGCLCs and BMP-rPGCLCs express representative pan-PGC markers (*Prdm14*, *Tfap2c*, *Blimp1*, and *Nanos3*) and pluripotency markers (*Pou5f1* and *Sox2*). In addition, T-rPGCLCs showed modest upregulation of some primitive streak (PS)/mesoderm markers (*Eomes*, *Cdx2*, *Tbx6*, *Mesp1*, *Mesp2*, *Foxc1*, and *Pdgfrb*) as well as late PGC markers (*Dazl*, *Ddx4*, and *Mael*) (Figures 1I and 2F). To confirm the function of T-rPGCLCs, we tested their capacity to undergo spermatogenesis after transplantation into the seminiferous tubules of *Prdm14* KO rats. After 9–12 weeks of transplantation, we observed some seminiferous tubules filled with AG-positive cells (Figure 1J; Table S1). IF analysis revealed the presence of AG and peanut agglutinin (PNA)-lectin double-positive round spermatids and sperm, indicating successful spermatogenesis originating from T-rPGCLCs (Figure 1K). Finally, we performed round spermatid injection (ROSI) using T-rPGCLC-derived spermatids and found that AG-positive round spermatids are capable of producing viable offspring (Figure 1L; Table S2). Taken together, we clearly demonstrate that T-rPGCLCs, induced in the absence of external BMP signals, are fully functional and equivalent to BMP-rPGCLCs.

(H) PCA showing the position of T-rPGCLCs within the developmental trajectory, integrating both *in vivo* and *in vitro* datasets. All transcriptome samples were obtained from 2 biologically independent experiments. The same applies below.

(I) Scatterplot showing correlation between BMP- and T-induced rPGCLCs.

(J) Testis 12 weeks after transplantation of T-rPGCLCs. Some seminiferous tubules positive for GFP indicate spermatogenesis originated from T-rPGCLCs. Scale bar is 5 mm.

(K) IF images of GFP-positive seminiferous tubules in Figure 1J. Scale bar is 100 μm.

(L) Offspring obtained by injection of T-rPGCLC-derived round spermatids into unfertilized rat oocytes. See also Figure S1 and Tables S1 and S2.

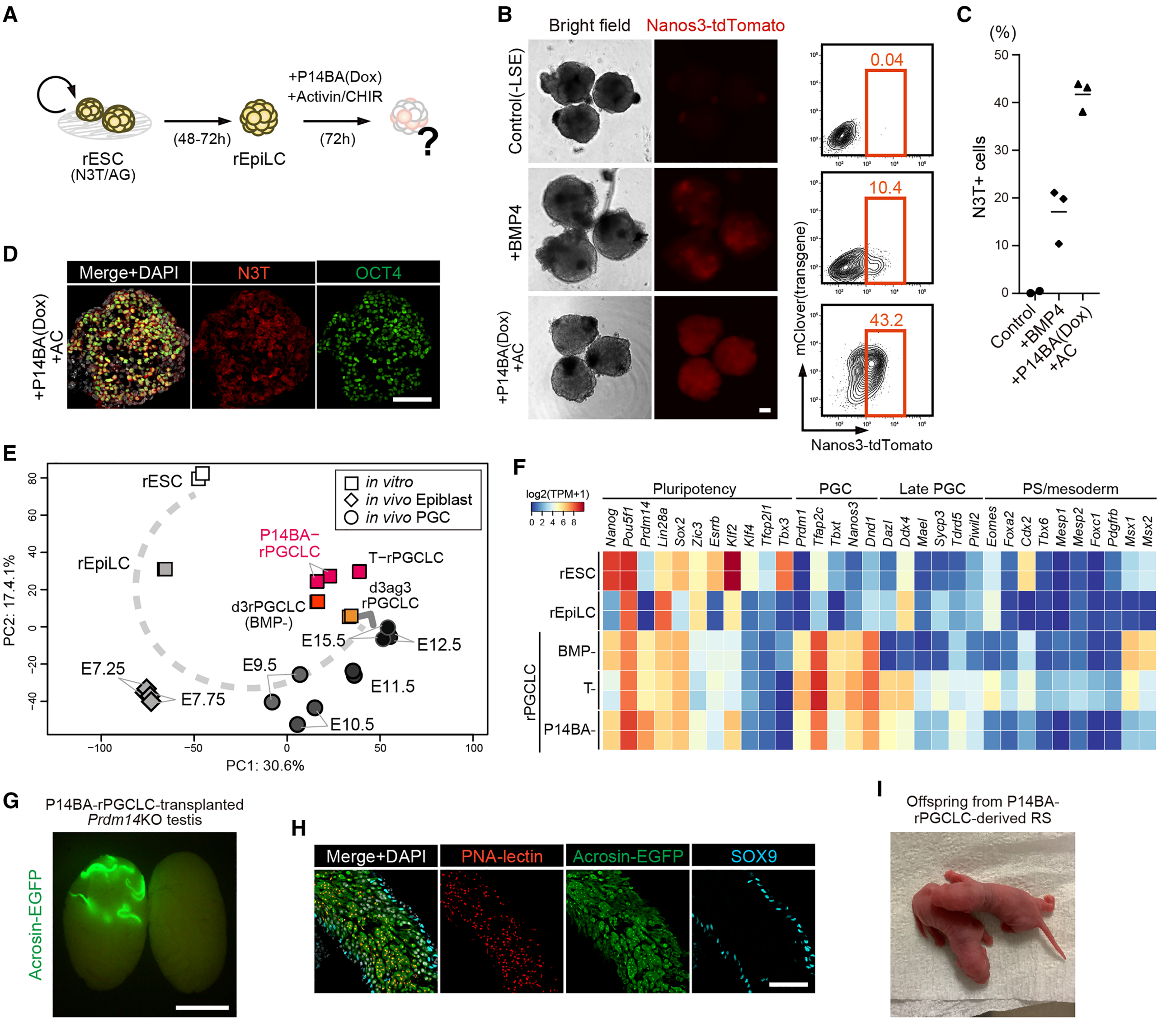

Figure 2. Supplementation of Activin and WNT signals allows *Prdm14/Blmip1/Ap2γ* to induce functional rPGCLC in the absence of BMP4

(A) Schematics of P14BA-mediated rPGCLC induction with supplementation of Activin-A and CHIR99021 (AC).
(B) Images and FACS patterns of day 3 rPGCLCs induced by only LSE, LSE plus BMP4, and LSE plus P14BA by adding Dox with AC. Scale bar is 100 μm.
(C) Dot plot showing percentage of Nanos3-tdTomato (N3T)-positive cells in Figure 2B (n = 2–3 biologically independent experiments).
(D) IF images of day 3 rPGCLCs induced by P14BA with AC. Scale bar is 100 μm.
(E) PCA showing the position of P14BA-rPGCLCs within the developmental trajectory, integrating both *in vivo* and *in vitro* datasets.
(F) Heatmap of representative gene expression of indicated samples.
(G) Testis 12 weeks after transplantation of P14BA-rPGCLCs. Scale bar is 5 mm.
(H) IF images of GFP-positive seminiferous tubules in Figure 2G. Scale bar is 100 μm.
(I) Offspring obtained by injection of P14BA-rPGCLC-derived round spermatids into unfertilized rat oocytes.
See also Figures S2 and S3 and Tables S1 and S2.

In mice, *Tbxt* is known to directly activate the expression of the PGC specifiers such as *Prdm14* and *Blimp1*, as well as mesodermal genes (Aramaki et al., 2013). Indeed, in rats, exogenous activation of *Tbxt* in rEpiLCs similarly led to a rapid (∼16 h) upregulation of *Prdm14* and *Blimp1* as well as endogenous *Tbxt* and the mesodermal gene *Cdx2*

(Figure S1H). Since extraembryonic mesodermal cells also express BMP4 (Lawson et al., 1999), we added the BMP signaling inhibitor LDN-193189 (LDN) during BMP- or T-rPGCLC induction to eliminate any indirect effects from mesoderm-derived BMP. As expected, BMP-induced rPGCLC induction was almost completely inhibited in the presence of LDN (Figure S1I). By contrast, overexpression of *Tbxt* in the presence of LDN reduced (~50%), but did not completely abolish rPGCLC induction (Figure S1I). This suggests that T-rPGCLCs comprises cells directly induced by *Tbxt* and others indirectly induced via *Tbxt*-mediated mesodermal BMP4 signaling.

Induction of rat PGCLCs by *Prdm14/Blimp1/Ap2γ* requires concomitant activation of signals for PS fate

We then asked why P14 or P14BA, downstream targets of *Tbxt*, are unable to initiate the germline program in rats, unlike in mice (Nakaki et al., 2013). Since rodent PGCs are specified in the posterior epiblast, where PS formation occurs, we hypothesize that activation of PS signals, combined with the enforced expression of these PGC specifiers, might induce rPGCLCs. In mice, Activin and WNT signals are essential for the induction of PS fate and for PGC specification (Aramaki et al., 2013; Gadue et al., 2006; Kobayashi et al., 2017; Loh et al., 2014; Ohinata et al., 2009). We confirmed that adding inhibitors against both canonical WNT signal and WNT secretion (XAV939, IWP-2) or an inhibitor of transforming growth factor β (TGF-β) Type I Receptor/ALK5 (SB-431542) nearly completely abrogated BMP-rPGCLC induction (Figure S2A), suggesting that the role of these signals in PGC specification are well conserved between mouse and rat. To activate the signaling pathway, we used Activin-A and CHIR99021 (AC), an inhibitor of glycogen synthase kinase-3 (GSK3) to activate canonical WNT pathways (Figure 2A). In the presence of AC, overexpression of P14BA efficiently induced N3T-positive rPGCLCs (P14BA-rPGCLCs; Figures 2B–2D), even in the absence of LSE cytokines. We confirmed that efficient induction of rPGCLCs, in the presence of AC, also can be achieved using a polycistronic vector (Figures S2B–S2D). This result is consistent in an independent cell line (rN3TAG#3) carrying the same transgenes (Figures S2E and S2F). Adding either Activin-A or CHIR99021 individually had a subtle effect on rPGCLC induction (Figures S2C and S2D), suggesting their synergistic role in enhancing rPGCLC induction, similar to PS fate induction as shown previously (Gadue et al., 2006). P14 or BA alone could also induce N3T-positive cells in the presence of AC, although with lower efficiency than P14BA (Figures S2G–S2J), suggesting a synergistic effect of the tripartite PGC specifiers (Magnusdottir et al., 2013; Nakaki et al., 2013). The transcriptome of P14BA-rPGCLCs is highly similar to that of BMP- and T-rPGCLCs (Figures 2E, S3A, and S3B). Like T-rPGCLCs, P14BA-rPGCLCs exhibit an overall similar gene expression pattern comparable to BMP-rPGCLCs, except for a modest upregulation of late PGC markers (Figure 2F). While T-rPGCLCs show a slight upregulation of some PS/mesodermal genes, P14BA-rPGCLC do not show this upregulation and instead further downregulate certain genes (*Cdx2*, *Msx1*, and *Msx2*) (Figure 2F). Thus, despite AC supplementation, which can induce PS fate, the subsequent or concomitant activation of the three PGC specifiers suppresses the somatic program, as shown in mice (Kurimoto et al., 2008). Functionally, P14BA-rPGCLCs transplanted into *Prdm14* KO neonatal testis were able to reconstitute spermatogenesis (Figures 2G and 2H; Table S1). Furthermore, isolated P14BA-rPGCLC-derived round spermatids contributed to the generation of viable offspring via ROSI (Figure 2I; Table S2), suggesting that P14BA-rPGCLCs are fully functional, similar to BMP-rPGCLCs. Overall, AC supplementation, which drives rEpiLCs toward a PS fate, promotes the induction of functional rPGCLCs through the activation of P14BA.

Etv4, identified as a downstream target of Activin and WNT signals, acts cooperatively with PGC specifiers

Since AC is known to upregulate PS genes, including *Tbxt*, we speculated that endogenous *Tbxt* activated by AC, together with exogenous P14BA led to the successful induction of rPGCLCs. To test this hypothesis, we disrupted *Tbxt* in P14BA-inducible rESCs (TKO), and examined whether P14BA can induce rPGCLCs in the presence of AC without *Tbxt* (Figures 3A and 3B). Consistent with previous observations in mice, two independent lines (#8, #13) of TKO rESCs formed rEpiLC normally, but failed to induce rPGCLCs in response to BMP signaling (Figure 3C). Unexpectedly, however, P14BA with AC was able to induce rPGCLCs with nearly similar efficiency as the WT control, even in the absence of *Tbxt* (Figure 3C). Thus, T-independent P14BA-rPGCLC induction implies the existence of other factor(s) that may act as downstream targets of AC, facilitating rPGCLC induction.

To identify the factor(s) regulated by WNT and Activin signals, we conducted a transcriptome analysis to examine differentially expressed genes 24 h after the activation of P14BA, based on the presence or absence of AC supplementation (Figure 4A). To exclude the influence of *Tbxt,* which is not required for P14BA-rPGCLC induction, we used wild-type (WT) and TKO rESC lines with a P14BA-inducible system. *Tbxt* was upregulated upon adding AC in WT but not TKO cells, at 24 h, indicating that *Tbxt* is a downstream target of AC, similar to its role in other mammals (Gadue et al., 2006; Kobayashi et al., 2017; Loh et al., 2014). Among the 26 genes upregulated both in WT and TKO ($Log2FC > 2$, $p < 0.05$, Figure 4B), we focused on five TFs (*Etv4*, *Foxi3*, *Hnf1b*, *Nkx2.1*, and *Pitx2*) and investigated whether their

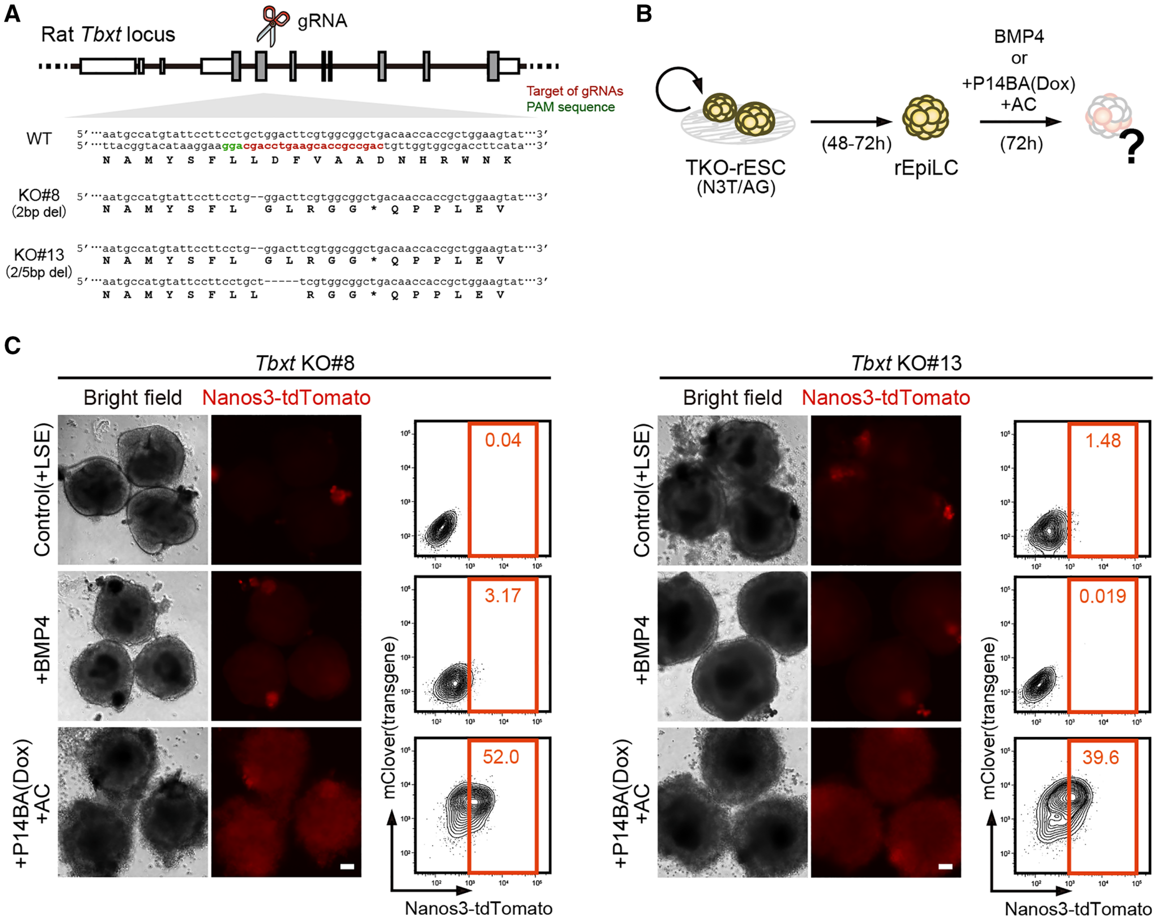

Figure 3. *Tbxt* is not necessary for induction of rPGCLCs by *Prdm14/Blmip1/Ap2γ* with Activin and WNT signals
(A) Strategy to generate TKO rESC lines by CRISPR-Cas9 system and their genotype (#8 and #13).
(B) Experimental design of induction of rPGCLC from TKO rESC lines.
(C) Images and FACS patterns of TKO day 3 rPGCLCs induced by only LSE, LSE plus BMP4, and LSE plus P14BA by adding Dox with AC. Results of 2 independent clones generated in Figure 3A are shown. Scale bar is 100 μm.

simultaneous activation, along with P14BA, could induce rPGCLCs in the absence of AC. Among the five candidate genes, we found that *Etv4* and *Hnf1b* could induce N3T-positive cells (Figures 4C and 4D). We further tested whether together, *Etv4* and *Hnf1b* more efficiently induced rPGCLCs, but no synergistic effect was observed (Figure S3E). Since the addition of *Hnf1b* had a modest effect in rPGCLC induction compared to *Etv4*, we focused further on the role of *Etv4*. Use of rN3TAG#3 cell line with P14BA and Etv4 transgenes shows consistent results (Figures S3C and S3D). Interestingly, *Etv4,* but not *Hnf1b,* is upregulated in T-rPGCLCs. We also confirmed that *Etv4* is modestly but rapidly (∼16 h) upregulated after activation of exogenous *Tbxt* (Figure S3F), suggesting that *Tbxt* likely induces *Blimp1, Prdm14,* and *Etv4,* which then work together to promote rPGCLCs induction, even in the absence of both BMP4 and AC. *Etv4* is a known downstream target of glial cell line-derived neurotrophic factor (GDNF) signaling in kidney development (Lu et al., 2009) and fibroblast growth factor (FGF) signaling in limb development (Mao et al., 2009). It plays a critical role in FGF-extracellular signal regulated kinase (ERK) signaling in pluripotent cells (Akagi et al., 2015; Simon et al., 2024; Yang et al., 2024). However, during P14BA-rPGCLC induction, replacing AC with FGF2 fails to induce PGCLCs (Figure S3G), suggesting a context-dependent relationship between external signals and transcriptional regulators. Finally, as a proof of concept, we confirmed that P14BA plus *Etv4*-induced rPGCLCs (P14BA + E-rPGCLCs) can reconstitute spermatogenesis (Figures 4E and 4F; Table S1), resulting in the birth of viable offspring (Figure 4G; Table S2). This result suggests that *Etv4* acts as a downstream target of AC in driving the induction of fully functional rPGCLCs (Figure 4I).

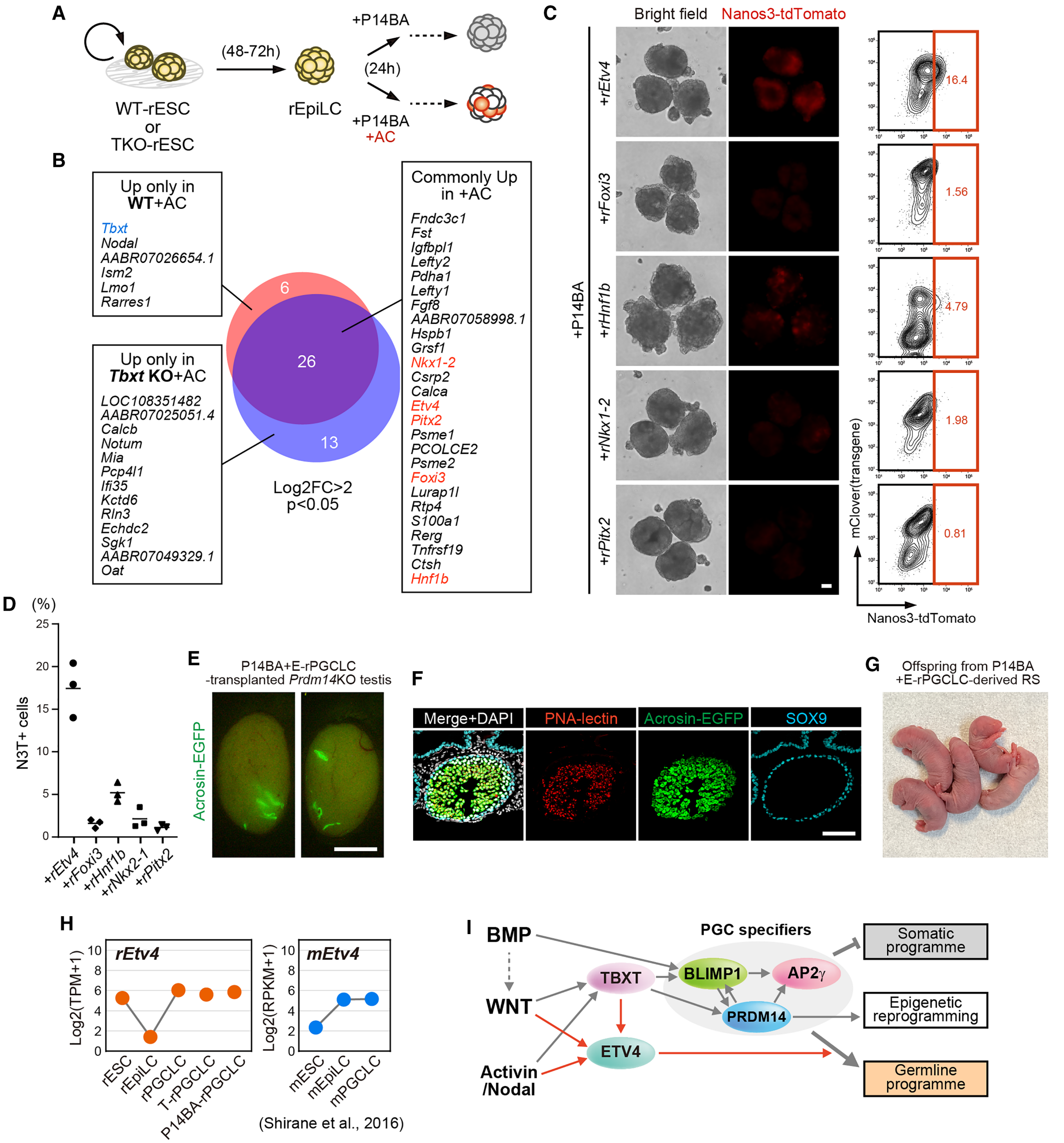

Figure 4. Candidate screening identifies *Etv4* acting cooperatively with *Prdm14/Blimp1/Ap2γ* to induce functional rPGCLCs

(A) Schematics to identify downstream target(s) of Activin and WNT signals during rPGCLC induction. At 24 h post-induction with Dox, with or without AC, cells were collected and subjected to RNA-seq analysis.

(B) Venn diagram showing genes upregulated in the presence of AC. Twenty six genes are commonly upregulated between WT and TKO.

(C) Images and FACS patterns of day 3 rPGCLCs induced by P14BA together with candidate transcription factors. Scale bar is 100 μm.

(D) Dot plot showing percentage of Nanos3-tdTomato (N3T)-positive cells in Figure 4C ($n = 3$ biologically independent experiments).

(E) Testis 12 weeks after transplantation of P14BA+E-rPGCLCs. Scale bar is 5 mm.

(F) IF images of GFP-positive seminiferous tubules in Figure 2G. Scale bar is 100 μm.

(legend continued on next page)

During BMP-rPGCLC induction, we observed that *Etv4* is temporally downregulated in rEpiLCs but re-activated in rPGCLCs (Figure 4H). Interestingly, in mice, *Etv4* expression is modestly upregulated in mEpiLCs compared with mESCs, and is maintained after BMP stimulation (Figure 4H). This may explain why P14BA is sufficient to induce PGCLCs in mice but not in rats. Among the erythroblast transformation specific (ETS) translocation variant (ETV) family members, *Etv4* is a member of the PEA3 subfamily of ETS transcription factors along with *Etv1* and *Etv5*. *Etv5* is highly expressed in PSCs, and is considered functionally redundant to *Etv4* (Akagi et al., 2015; Kalkan et al., 2019). During the stepwise induction of rPGCLC from rESCs, both *Etv5* and *Etv1* are consistently expressed at similar levels throughout the transition (Figure S3H). Since *Etv4* is upregulated in PGCLCs compared to EpiLCs (Figure 4H), our study suggests that *Etv4* may play a specific role in germline specification. In conclusion, we identified that *Etv4* cooperates with the three PGC specifiers to induce PGC fate in rats, a mechanism that may have been overlooked in mice but was successfully uncovered using our *in vitro* rat system.

DISCUSSION

In this study, we demonstrate the induction of PGCLCs from EpiLCs through the overexpression of transcriptional regulator(s) in rats, in the absence of BMP4. We found that T-, P14BA-, and P14BA + E-induced rPGCLCs are functionally equivalent to BMP4-induced rPGCLCs in their efficiency to reconstitute spermatogenesis following transplantation (Oikawa et al., 2022). Although a previous report demonstrated that *Tbxt* could induce PGC fate in mice, the subsequent developmental potential of T-induced mPGCLCs after specification has remained unclear (Aramaki et al., 2013). Our data using the rat model clearly demonstrates that T-induced rPGCLCs can normally contribute to gametogenesis, resulting in the birth of viable offspring via ROSI. It should be noted, however, that T-rPGCLCs include rPGCLCs induced by mesoderm-derived BMP. In mice, lower levels of *Tbxt* promote PGC fate, whereas higher levels promote mesodermal fate (Aramaki et al., 2021). Thus, optimal *Tbxt* dosage may be critical for direct induction of rPGCLCs.

Overexpression of transcription factor(s) in bulk selected populations can lead to ambiguous boundaries between the negative and positive populations, likely due to variation in transgene expression. This variability could be due to differences in transgene introduction and subsequent antibiotic selection, resulting in heterogeneous expression levels. To achieve efficient rPGCLC induction, it is crucial to optimize the dosage of each transcription factor. Nevertheless upon T-, P14BA-, and P14BA + E-rPGCLC transplantation into seminiferous tubules, Dox or Dex activation of exogenous transgenes was no longer required. Therefore, once the transcriptional regulatory network to specify PGC fate is established by the exogenous transgenes, subsequent PGC development and gametogenesis proceed independently of the transgenes, cell-autonomously.

PGCLC induction with the three PGC specifiers, *Prdm14*, *Blimp1, and Ap2γ*, requires additional Activin and WNT signals in rats. In mice, mEpiLCs are induced as an adherent monolayer, and after dissociation, formation of aggregates activates *Tbxt* (Okashita et al., 2016), likely via the canonical WNT signal pathway. Indeed, *Wnt3* is modestly upregulated in EpiLCs in mice (Hayashi et al., 2011; Nakaki et al., 2013). Since the three PGC specifiers are exogenously activated by adding Dox after aggregate formation in mice, they may function on a state biased toward PS fate. In rats, however, rPGCLCs are induced by simply transferring rEpiLC aggregates to rPGCLC medium without dissociation (Oikawa et al., 2022), this approach may not lead to the induction of PS fate. While BMP4 can induce rPGCLCs, likely through the activation of WNT3 in the epiblast (Aramaki et al., 2013; Ben-Haim et al., 2006), for P14BA-mediated rPGCLC induction, the exogenous activation of WNT and Activin/Nodal signals may be crucial for the appropriate response to P14BA for the establishment of PGC fate in the absence of BMP signal.

From our candidate screening, we identified *Etv4* as a downstream target of Activin and WNT signals. A recent report showed that the expression of *ETV4* varies depending on the size of human PSC colonies and its expression connects cell crowding with lineage specification (Yang et al., 2024). ETV4 positive cells located at the colony edges exhibit mesendodermal fate, while ETV4 negative areas in the colony center are associated with neuroectodermal fate (Yang et al., 2024). Since human PSCs gain competency for PGC fate during their progression toward mesendodermal fate (Kobayashi et al., 2017; Vijayakumar et al., 2023), *ETV4* may play a conserved role in PGC competency across species. In mice, *Etv4* KO animals are sterile due to

(G) Offspring obtained by injection of P14BA+E-rPGCLC-derived round spermatids into unfertilized rat oocytes.
(H) Expression patterns of *Etv4* in rats (this study) and mice (Shirane et al., 2016).
(I) Summary of this study. The gray arrow indicates a mechanism known in mouse model, and the red arrow indicates a potential mechanism uncovered in this study. The three PGC specifiers, enclosed by a gray circle, act together with *Etv4* to activate the germline program.
See also Figure S3 and Tables S1 and S2.

abnormal spermiogenesis, but not due to defects in PGC specification (Laing et al., 2000). Another study demonstrated that self-renewing formative mPSCs can generate PGCLCs in the absence of both *Etv4* and *Etv5*, suggesting that other factor(s) may compensate for the function of *Etv4* during PGC specification (Kinoshita et al., 2021). Since the efficiency of rPGCLC induction by P14BA + E is still lower than that of P14BA with AC, additional factor (s) may also be involved in regulating PGC specification.

The rat as a model can be leveraged for its similarities and differences to the mouse model, making it valuable for testing whether findings in mice are conserved across species or specific to mice. For instance, in our study, validating mouse results in the rat model led to the identification of a novel transcriptional regulator, *Etv4*, which cooperates with the three previously identified PGC specifiers to induce PGC fate. Similar to PSC culture and PGCLC induction, capturing and reconstituting rat embryonic development often requires more stringent conditions with less redundancy compared to mice. Thus, further investigation using the rat model, to review and validate findings in mice, as demonstrated in our study will provide deeper mechanistic insights into early embryo and germline development. This could ultimately pave the way for the development of widely applicable *in vitro* gametogenesis techniques in the future.

METHODS

Culture and genetic manipulation of rESCs

Male rESC lines harboring Nanos3-tdTomato reporter and Acrosin-EGFP reporter (rN3TAG#2, #3) was derived previously (Oikawa et al., 2022). The rESCs were routinely cultured in rESC medium: N2B27 medium containing 2i (PD0325901, 1 μM, Axon, Groeningen, The Netherlands; CHIR99021, 3 μM, Axon) and rat LIF (1,000 U/mL, Merck Millipore, MA). All the components of N2B27 were purchased from Thermo Fisher Scientific according to published protocol (Hirabayashi et al., 2019; Oikawa et al., 2024). The rESCs were passaged by 0.25% Trypsin-EDTA (Thermo Fisher Scientific) every 2–3 days according to our published protocol (Hirabayashi et al., 2019; Oikawa et al., 2024). For introducing exogenous transgenes, PiggyBac vectors containing Dox-inducible or Dex-inducible systems previously developed (Kobayashi et al., 2017) were used accordingly (Figures 1A and S2A). All rat cDNAs were amplified by PCR using PrimeSTAR MAX DNA polymerase (Takara Bio, Shiga, Japan) or KOD One DNA polymerase (Toyobo, Osaka, Japan) according to the manufacturer's protocol. For generating Tbxt KO rESCs, gRNA sequence described in Figure 3A is subcloned into eSpCas9(1.1) (Addgene #71814) CRISPR/Cas9 vector. PiggyBac vectors with PBase plasmid or CRISPR/Cas9 vector were transfected into rESCs by reverse transfection method. For PiggyBAC vectors, the transfected rESCs were seeded on a Neomycin and Puromycin resistance feeder generated in-house (Ohtsuka et al., 2015), and 48 h later, 0.8–1.0 μg/mL puromycin (Sigma-Aldrich, MO) and/or 200 μg/mL G418 (Sigma-Aldrich) were added to the culture medium for selection.

Induction of rPGCLCs

The protocol is essentially the same as our published one (Oikawa et al., 2022, 2024). rESCs were dissociated using 0.25% Trypsin-EDTA and harvested in 96-well Nunclon Sphera-Treated U-shaped microplate (Thermo Fisher Scientific) containing 100 μL of rEpiLC medium at 4×10^3 cells per well. rEpiLC medium composed of N2B27 medium containing Activin A (20 ng/mL, Peprotech), bFGF (12 ng/mL; Peprotech), and 1% KSR (Thermo Fisher Scientific) was prepared freshly on the day of use. On day 1 of rEpiLC induction, each well was topped up with 100 μL of fresh medium. On day 2, half volume of the medium was replaced with fresh medium. On day 2–3 after induction, the rEpiLCs were washed in PBS containing 3% fetal bovine serum (FBS) or N2B27 containing 5% KSR using a glass capillary. Then, rEpiLCs were transferred into a well of 96-well Nunclon Sphera-Treated U-shaped microplate containing 100 μL of freshly made rPGCLC medium composed of N2B27 medium containing BMP4 (500 ng/mL; Peprotech), rat LIF (1000 U/mL; Merck Millipore), mSCF (100 ng/mL; R&D systems), mEGF (50 ng/mL; R&D systems), and 5% KSR. For induction of exogenous transgenes, 100 μM Dex (Sigma), or 1 μg/mL Dox (Sigma) were added to the medium instead of BMP4 or other cytokines. To test signaling inhibitors, TGF-β Type I Receptor inhibitor SB-431542 (10 μM; Selleck Chemicals, TX), WNT inhibitors IWP-2 (1 μM; Tocris Bioscience, Bristol, UK) and XAV939 (5 μM; Cayman Chemical; MI), or LDN-193189 (0.5 μM; Tocris Bioscience) were used, respectively. At day 3 after rPGCLC induction, rPGCLCs were subjected to the downstream analysis. The morphology and fluorescent of rPGCLCs were observed using BZ-X810 (Keyence, Osaka, Japan).

RESOURCE AVAILABILITY

Lead contact

Further information and requests for resources and reagents should be directed to the lead contact, Toshihiro Kobayashi (tkoba@nips.ac.jp).

Materials availability

Unique reagents generated in this study are available from the lead contact with a materials transfer agreement.

Data and code availability

- This paper analyzes existing, publicly available data. These accession numbers for the datasets are listed in the key resources table. RNA-seq data had been deposited in the Sequence Read Archive (SRA) under BioProject ID: PRJNA1199573.
- Any additional information required to reanalyze the data reported in this paper is available from the lead contact upon request.

ACKNOWLEDGMENTS

We thank members of the Kobayashi lab and Hirabayashi lab, particularly Keiko Yamauchi and Fumika Yoshida, for technical assistance, and Minako Ohnishi for secretarial support. We also thank Dr. Roopsha Sengupta for editing and providing critical input to the manuscript. Flow cytometry was performed in the IMSUT FACS Core laboratory. This work was supported by JSPS KAKENHI 18H02367, 20H03167, 23K20043, 24K01944, and 25H01349 to T.K.; 18H05544 to T.K. and K.K.; 19K23711 to M. O.; 21H02382 to H. Kobayashi; 21J21849 to K.I.; 21J21849, AMED JP18bm0704022, and JP22bm1123008 to T.K.; JST JPMJFR233J to T.K.; Cooperative Study Program 24NIPS310 of NIPS to T.K.; and grants from The Sumitomo Foundation 210348 to T.K., Kato Memorial Bioscience Foundation to T.K., and Daiichi Sankyo Foundation of Life Science to T.K.

AUTHOR CONTRIBUTIONS

Conceptualization: M.O. and T.K.; methodology: M.O., H. Kojima, K.I., H. Kobayashi, K.N., M.S., T. Yamamoto, M.H., and T.K.; formal analysis: M.O. and T.K.; investigation: M.O. and T.K.; visualization: M.O. and T.K.; writing: M.O. and T.K.; supervision: T. Yamaguchi, K.K., and T.K.; funding acquisition: T.K.

DECLARATION OF INTERESTS

The authors declare no competing interests.

SUPPLEMENTAL INFORMATION

Supplemental information can be found online at https://doi.org/10.1016/j.stemcr.2025.102599.

Received: February 9, 2025
Revised: July 11, 2025
Accepted: July 14, 2025
Published: August 12, 2025

REFERENCES

Akagi, T., Kuure, S., Uranishi, K., Koide, H., Costantini, F., and Yokota, T. (2015). ETS-related transcription factors ETV4 and ETV5 are involved in proliferation and induction of differentiation-associated genes in embryonic stem (ES) cells. J. Biol. Chem. *290*, 22460–22473.

Aramaki, S., Hayashi, K., Kurimoto, K., Ohta, H., Yabuta, Y., Iwanari, H., Mochizuki, Y., Hamakubo, T., Kato, Y., Shirahige, K., and Saitou, M. (2013). A mesodermal factor, T, specifies mouse germ cell fate by directly activating germline determinants. Dev. Cell *27*, 516–529.

Aramaki, S., Kagiwada, S., Wu, G., Obridge, D., Adachi, K., Kutejova, E., Lickert, H., Hübner, K., and Schöler, H.R. (2021). Residual pluripotency is required for inductive germ cell segregation. EMBO Rep. *22*, e52553.

Ben-Haim, N., Lu, C., Guzman-Ayala, M., Pescatore, L., Mesnard, D., Bischofberger, M., Naef, F., Robertson, E.J., and Constam, D. B. (2006). The nodal precursor acting via activin receptors induces mesoderm by maintaining a source of its convertases and BMP4. Dev. Cell *11*, 313–323.

Gadue, P., Huber, T.L., Paddison, P.J., and Keller, G.M. (2006). Wnt and TGF-beta signaling are required for the induction of an *in vitro* model of primitive streak formation using embryonic stem cells. Proc. Natl. Acad. Sci. USA *103*, 16806–16811.

Hayashi, K., Ohta, H., Kurimoto, K., Aramaki, S., and Saitou, M. (2011). Reconstitution of the mouse germ cell specification pathway in culture by pluripotent stem cells. Cell *146*, 519–532.

Hirabayashi, M., Takizawa, A., and Hochi, S. (2019). Embryonic Stem Cells and Gene Manipulation in Rat. Methods Mol. Biol. *2018*, 115–130.

Kalkan, T., Bornelöv, S., Mulas, C., Diamanti, E., Lohoff, T., Ralser, M., Middelkamp, S., Lombard, P., Nichols, J., and Smith, A. (2019). Complementary Activity of ETV5, RBPJ, and TCF3 Drives Formative Transition from Naive Pluripotency. Cell Stem Cell *24*, 785–801.e7.

Kinoshita, M., Barber, M., Mansfield, W., Cui, Y., Spindlow, D., Stirparo, G.G., Dietmann, S., Nichols, J., and Smith, A. (2021). Capture of Mouse and Human Stem Cells with Features of Formative Pluripotency. Cell Stem Cell *28*, 453–471.e8.

Kobayashi, T., Goto, T., Oikawa, M., Sanbo, M., Yoshida, F., Terada, R., Niizeki, N., Kajitani, N., Kazuki, K., Kazuki, Y., et al. (2021). Blastocyst complementation using Prdm14-deficient rats enables efficient germline transmission and generation of functional mouse spermatids in rats. Nat. Commun. *12*, 1328.

Kobayashi, T., Kobayashi, H., Goto, T., Takashima, T., Oikawa, M., Ikeda, H., Terada, R., Yoshida, F., Sanbo, M., Nakauchi, H., et al. (2020). Germline development in rat revealed by visualization and deletion of Prdm14. Development *147*, dev183798.

Kobayashi, T., and Surani, M.A. (2018). On the origin of the human germline. Development *145*, dev150433.

Kobayashi, T., Zhang, H., Tang, W.W.C., Irie, N., Withey, S., Klisch, D., Sybirna, A., Dietmann, S., Contreras, D.A., Webb, R., et al. (2017). Principles of early human development and germ cell program from conserved model systems. Nature *546*, 416–420.

Kojima, Y., Sasaki, K., Yokobayashi, S., Sakai, Y., Nakamura, T., Yabuta, Y., Nakaki, F., Nagaoka, S., Woltjen, K., Hotta, A., et al. (2017). Evolutionarily Distinctive Transcriptional and Signaling Programs Drive Human Germ Cell Lineage Specification from Pluripotent Stem Cells. Cell Stem Cell *21*, 517–532.e5.

Kojima, Y., Yamashiro, C., Murase, Y., Yabuta, Y., Okamoto, I., Iwatani, C., Tsuchiya, H., Nakaya, M., Tsukiyama, T., Nakamura, T., et al. (2021). GATA transcription factors, SOX17 and TFAP2C, drive the human germ-cell specification program. Life Sci. Alliance *4*, e202000974.

Kurimoto, K., Yabuta, Y., Ohinata, Y., Shigeta, M., Yamanaka, K., and Saitou, M. (2008). Complex genome-wide transcription dynamics orchestrated by Blimp1 for the specification of the germ cell lineage in mice. Genes Dev. *22*, 1617–1635.

Laing, M.A., Coonrod, S., Hinton, B.T., Downie, J.W., Tozer, R., Rudnicki, M.A., and Hassell, J.A. (2000). Male sexual dysfunction in mice bearing targeted mutant alleles of the PEA3 ets gene. Mol. Cell Biol. *20*, 9337–9345.

Lawson, K.A., Dunn, N.R., Roelen, B.A., Zeinstra, L.M., Davis, A.M., Wright, C.V., Korving, J.P., and Hogan, B.L. (1999). Bmp4 is required for the generation of primordial germ cells in the mouse embryo. Genes Dev. *13*, 424–436.

Loh, K.M., Ang, L.T., Zhang, J., Kumar, V., Ang, J., Auyeong, J.Q., Lee, K.L., Choo, S.H., Lim, C.Y.Y., Nichane, M., et al. (2014). Efficient endoderm induction from human pluripotent stem cells by logically directing signals controlling lineage bifurcations. Cell Stem Cell *14*, 237–252.

Lu, B.C., Cebrian, C., Chi, X., Kuure, S., Kuo, R., Bates, C.M., Arber, S., Hassell, J., MacNeil, L., Hoshi, M., et al. (2009). Etv4 and Etv5 are required downstream of GDNF and Ret for kidney branching morphogenesis. Nat. Genet. *41*, 1295–1302.

Magnusdottir, E., Dietmann, S., Murakami, K., Gunesdogan, U., Tang, F., Bao, S., Diamanti, E., Lao, K., Gottgens, B., and Azim Surani, M. (2013). A tripartite transcription factor network regulates primordial germ cell specification in mice. Nat. Cell Biol. *15*, 905–915.

Mao, J., McGlinn, E., Huang, P., Tabin, C.J., and McMahon, A.P. (2009). Fgf-dependent Etv4/5 activity is required for posterior restriction of Sonic Hedgehog and promoting outgrowth of the vertebrate limb. Dev. Cell *16*, 600–606.

Nakaki, F., Hayashi, K., Ohta, H., Kurimoto, K., Yabuta, Y., and Saitou, M. (2013). Induction of mouse germ-cell fate by transcription factors *in vitro*. Nature *501*, 222–226.

Ohinata, Y., Ohta, H., Shigeta, M., Yamanaka, K., Wakayama, T., and Saitou, M. (2009). A signaling principle for the specification of the germ cell lineage in mice. Cell *137*, 571–584.

Ohinata, Y., Payer, B., O'Carroll, D., Ancelin, K., Ono, Y., Sano, M., Barton, S.C., Obukhanych, T., Nussenzweig, M., Tarakhovsky, A., et al. (2005). Blimp1 is a critical determinant of the germ cell lineage in mice. Nature *436*, 207–213.

Ohtsuka, M., Miura, H., Mochida, K., Hirose, M., Hasegawa, A., Ogura, A., Mizutani, R., Kimura, M., Isotani, A., Ikawa, M., et al. (2015). One-step generation of multiple transgenic mouse lines using an improved Pronuclear Injection-based Targeted Transgenesis (i-PITT). BMC Genom. *16*, 274.

Oikawa, M., Hirabayashi, M., and Kobayashi, T. (2024). Induction of Primordial Germ Cell-Like Cells from Rat Pluripotent Stem Cells. Methods Mol. Biol. *2770*, 99–111.

Oikawa, M., Kobayashi, H., Sanbo, M., Mizuno, N., Iwatsuki, K., Takashima, T., Yamauchi, K., Yoshida, F., Yamamoto, T., Shinohara, T., et al. (2022). Functional primordial germ cell-like cells from pluripotent stem cells in rats. Science *376*, 176–179.

Okashita, N., Suwa, Y., Nishimura, O., Sakashita, N., Kadota, M., Nagamatsu, G., Kawaguchi, M., Kashida, H., Nakajima, A., Tachibana, M., and Seki, Y. (2016). PRDM14 Drives OCT3/4 Recruitment via Active Demethylation in the Transition from Primed to Naive Pluripotency. Stem Cell Rep. *7*, 1072–1086.

Seki, Y. (2018). PRDM14 Is a Unique Epigenetic Regulator Stabilizing Transcriptional Networks for Pluripotency. Front. Cell Dev. Biol. *6*, 12.

Shirane, K., Kurimoto, K., Yabuta, Y., Yamaji, M., Satoh, J., Ito, S., Watanabe, A., Hayashi, K., Saitou, M., and Sasaki, H. (2016). Global Landscape and Regulatory Principles of DNA Methylation Reprogramming for Germ Cell Specification by Mouse Pluripotent Stem Cells. Dev. Cell *39*, 87–103.

Simon, C.S., Garg, V., Kuo, Y.-Y., Niakan, K.K., and Hadjantonakis, A.-K. (2024). ETV4 and ETV5 Orchestrate FGF-Mediated Lineage Specification and Epiblast Maturation during Early Mouse Development. Preprint at bioRxiv. https://doi.org/10.1101/2024.07.24.604964.

Tang, W.W.C., Castillo-Venzor, A., Gruhn, W.H., Kobayashi, T., Penfold, C.A., Morgan, M.D., Sun, D., Irie, N., and Surani, M.A. (2022). Sequential enhancer state remodelling defines human germline competence and specification. Nat. Cell Biol. *24*, 448–460.

Vijayakumar, S., Sala, R., Kang, G., Chen, A., Pablo, M.A., Adebayo, A.I., Cipriano, A., Fowler, J.L., Gomes, D.L., Ang, L.T., et al. (2023). Monolayer platform to generate and purify primordial germ-like cells *in vitro* provides insights into human germline specification. Nat. Commun. *14*, 5690.

Weber, S., Eckert, D., Nettersheim, D., Gillis, A.J.M., Schäfer, S., Kuckenberg, P., Ehlermann, J., Werling, U., Biermann, K., Looijenga, L.H.J., and Schorle, H. (2010). Critical function of AP-2 gamma/TCFAP2C in mouse embryonic germ cell maintenance. Biol. Reprod. *82*, 214–223.

Yamaji, M., Seki, Y., Kurimoto, K., Yabuta, Y., Yuasa, M., Shigeta, M., Yamanaka, K., Ohinata, Y., and Saitou, M. (2008). Critical function of Prdm14 for the establishment of the germ cell lineage in mice. Nat. Genet. *40*, 1016–1022.

Yang, S., Golkaram, M., Oh, S., Oh, Y., Cho, Y., Yoe, J., Ju, S., Lalli, M.A., Park, S.-Y., Lee, Y., and Jang, J. (2024). ETV4 is a mechanical transducer linking cell crowding dynamics to lineage specification. Nat. Cell Biol. *26*, 903–916.

Ying, Y., Qi, X., and Zhao, G.Q. (2001). Induction of primordial germ cells from murine epiblasts by synergistic action of BMP4 and BMP8B signaling pathways. Proc. Natl. Acad. Sci. USA *98*, 7858–7862.

Stem Cell Reports, Volume *20*

Supplemental Information

Transcription factor-mediated germ cell induction in rats reveals ETV4 cooperates with germline specifiers

Mami Oikawa, Hiroki Kojima, Hisato Kobayashi, Kenyu Iwatsuki, Hijiri Saito, Makoto Sanbo, Kazumi Nishioka, Tomoyuki Yamaguchi, Takuya Yamamoto, Kazuki Kurimoto, Masumi Hirabayashi, and Toshihiro Kobayashi

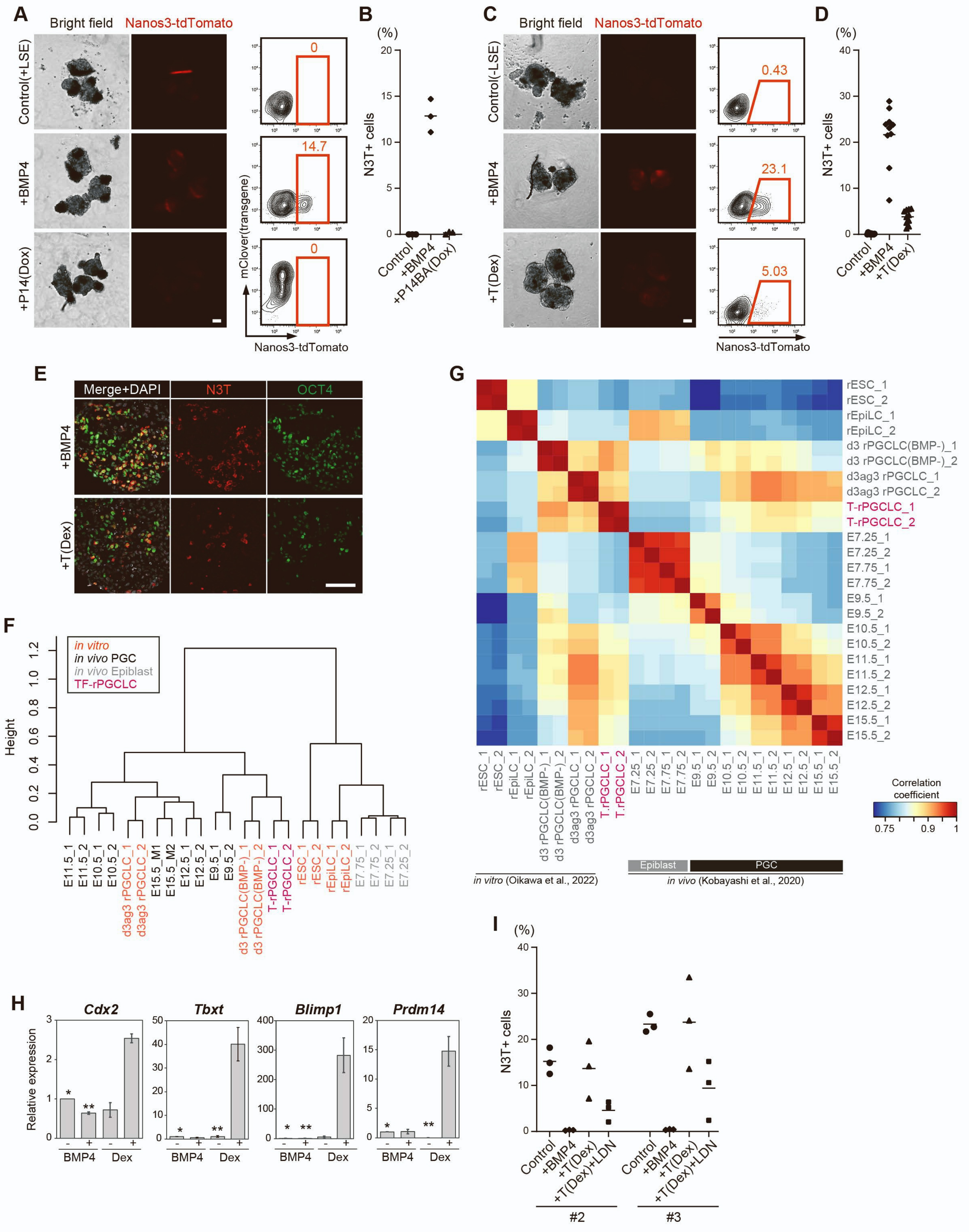

Oikawa et al., Figure S1

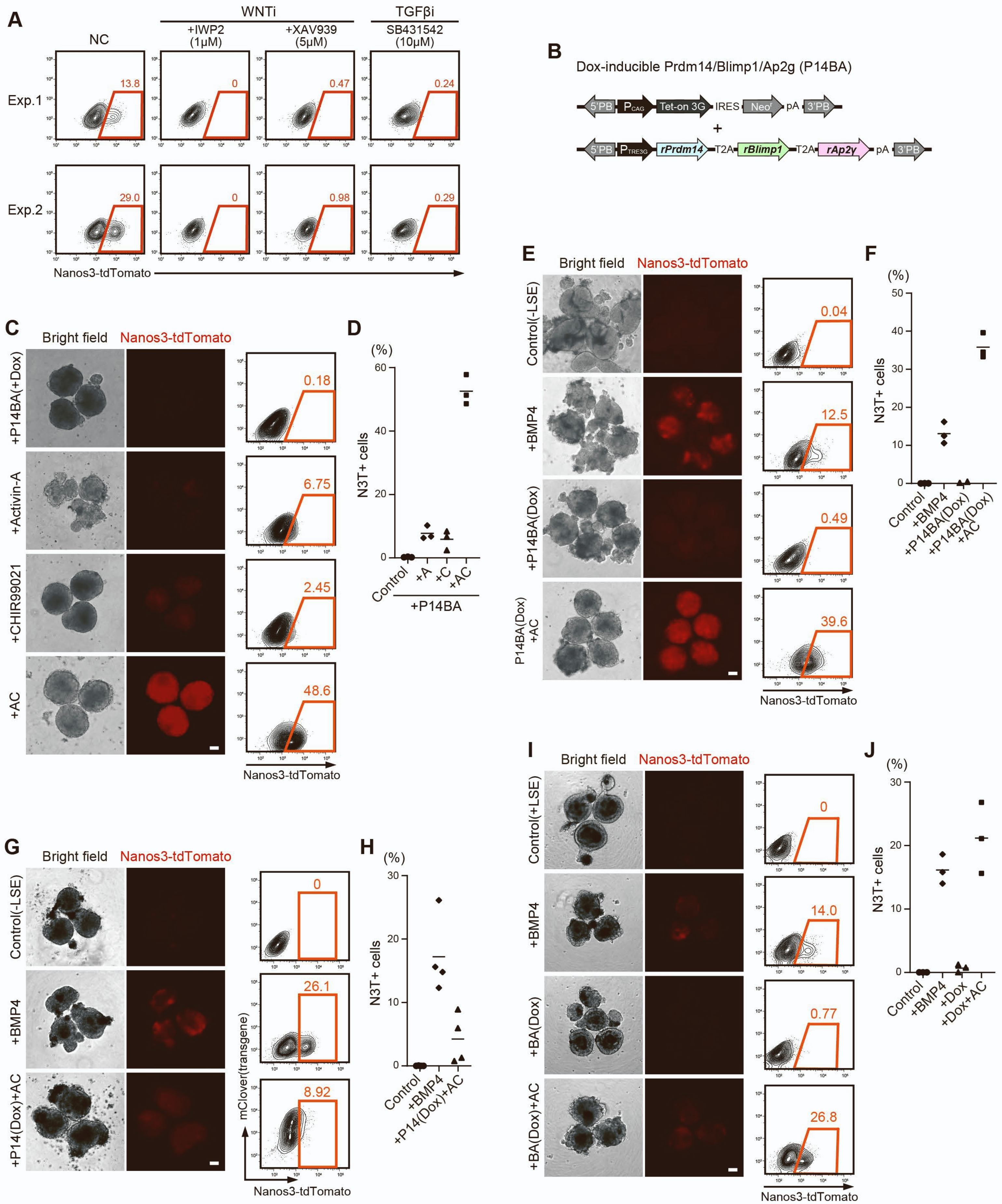

Oikawa et al., Figure S2

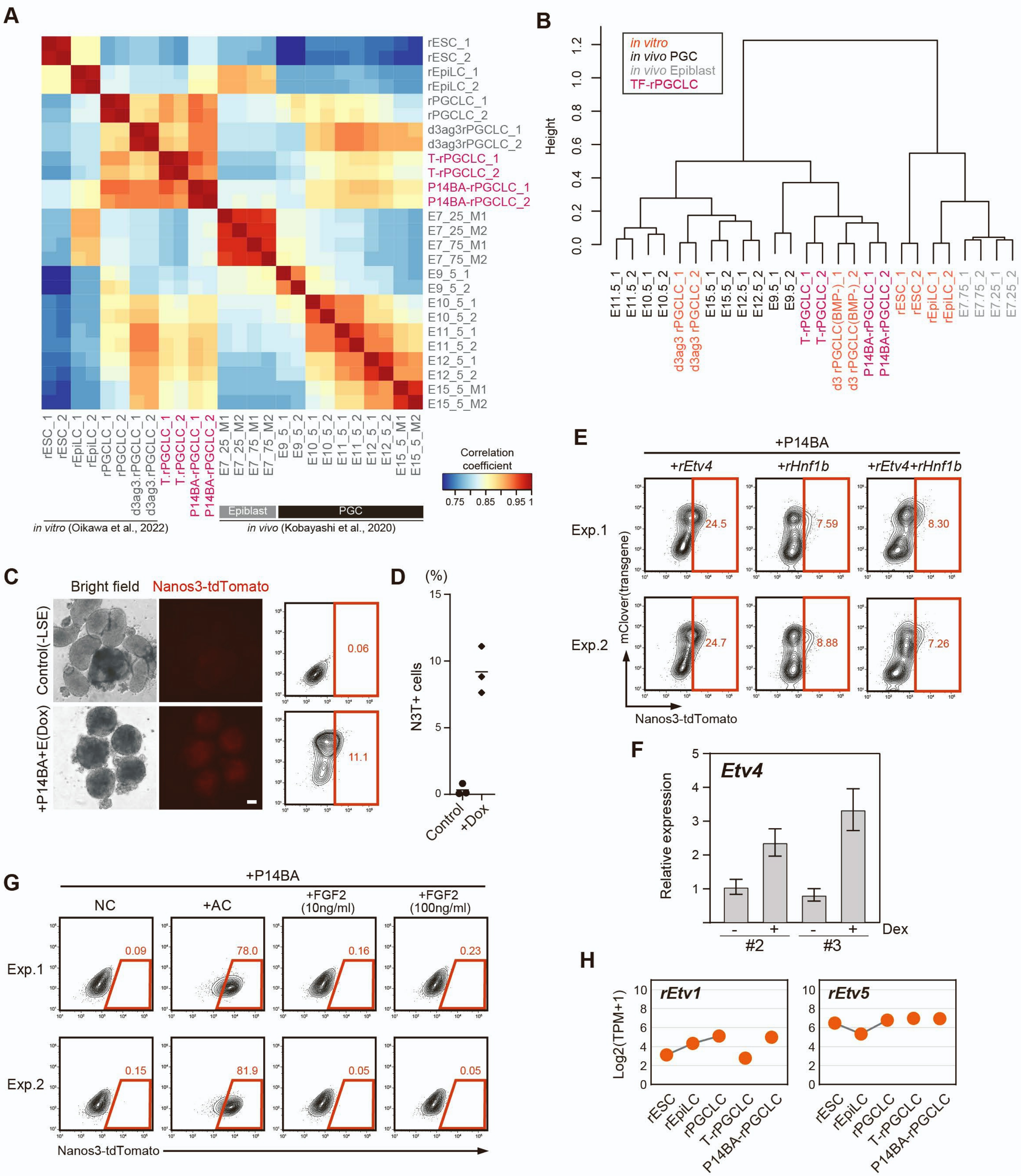

Oikawa et al., Figure S3

SUPPLEMENTARY FIGURE TITLES AND LEDENDS

Figure S1. Characterization of T-rPGCLC, related to Figure 1

(A) Images and FACS patterns of day 3 rPGCLCs induced by only LSE, LSE plus BMP4, and LSE plus P14 by adding Dox. Scale bar is 100 µm.

(B) Dot plot showing percentage of Nanos3-tdTomato (N3T) positive cells in **Figure S1A** (n = 3 biologically independent experiments).

(C) Images and FACS patterns of day 3 rPGCLCs induced by cytokine-free condition, BMP4, and T by adding Dex. Scale bar is 100 µm.

(D) Dot plot showing percentage of Nanos3-tdTomato (N3T) positive cells in **Figure S1C** (n = 10 biologically independent experiments).

(E) IF images of day 3 rPGCLCs induced by BMP4 or T. Scale bar is 100 µm.

(F) Hierarchical clustering among T-rPGCLCs and published in vitro/in vivo samples as indicated.

(G) Heatmap of the correlation coefficients among T-rPGCLCs and published in vitro/in vivo samples as indicated.

(H) RT-qPCR analysis of cells at 16 h after induction of rPGCLCs by BMP4 or exogenous *Tbxt* by Dex. Relative expression levels to BMP4 minus controls are shown. Values are normalized in reference to *rActb*. Bars represent averages of n = 3 biologically independent experiments. One or two asterisks indicate Ct values detected in only one or two samples due to low expression levels.

(I) Dot plot showing percentage of Nanos3-tdTomato (N3T) positive cells in BMP- and T-rPGCLCs with or without BMP inhibitor (n = 3 biologically independent experiments, 2 independent cell lines).

Figure S2. Characterization of P14BA-rPGCLC, related to Figure 2

(A) FACS patterns of day 3 rPGCLCs induced by BMP4 plus LSE with or without Activin receptor inhibitor, SB431542 (10uM), or WNT inhibitors, XAV939 (5uM) and/or IWP-2 (1uM) (n = 2 biologically independent experiments).

(B) A polycistronic gene-inducible system in this study; Dox-inducible *rPrdm14-T2A-rBlimp1-T2A-rAp2*γ.

(C) Images and FACS patterns of day 3 rPGCLCs induced by P14BA with or without Activin A and CHIR99021 as indicated. Scale bar is 100 µm.

(D) Dot plot showing percentage of Nanos3-tdTomato (N3T) positive cells in **Figure S2B** (n = 3 biologically independent experiments).

(E) Images and FACS patterns of day 3 rPGCLCs from rN3TAG#3 induced by P14BA. Scale bar is 100 µm.

(F) Dot plot showing percentage of Nanos3-tdTomato (N3T) positive cells in **Figure S2E** (n = 2-3 biologically independent experiments).

(G) Images and FACS patterns of day 3 rPGCLCs induced by cytokine free condition, BMP4, and P14 by adding Dox with AC. Scale bar is 100 µm.

(H) Dot plot showing percentage of Nanos3-tdTomato (N3T) positive cells in **Figure S2D** (n = 4 biologically independent experiments).

(I) Images and FACS patterns of day 3 rPGCLCs induced by only LSE, BMP4 plus LSE, and BA by adding Dox with or without AC. Scale bar is 100 µm.

(J) Dot plot showing percentage of Nanos3-tdTomato (N3T) positive cells in **Figure S2F** (n = 3 biologically independent experiments).

Figure S3. Effects of signals and transgenes for rPGCLC induction, related to Figure 2 and 4

(A) Hierarchical clustering among P14BA-rPGCLCs and published in vitro/in vivo samples as indicated.

(B) Heatmap of the correlation coefficients among P14BA-rPGCLCs and published in vitro/in vivo samples as indicated.

(C) Images and FACS patterns of day 3 rPGCLCs from rN3TAG#3 induced by P14BA+E. Scale bar is 100 µm.

(D) Dot plot showing percentage of Nanos3-tdTomato (N3T) positive cells in **Figure S3C** (n = 3 biologically independent experiments).

(E) FACS patterns of day 3 rPGCLCs induced by P14BA together with *rEtv4* and/or *Hnf1b*. (n = 2 biologically independent experiments)

(F) RT-qPCR analysis of cells at 16 h after induction of rPGCLCs by exogenous *Tbxt* by Dex. Relative expression levels to Dex minus control in rN3TAG#2 are shown. Values are normalized in reference to *rActb*. Bars represent averages of n = 2 biologically independent experiments.

(G) FACS patterns of day 3 rPGCLCs induced by P14BA with AC or FGF2.

(H) Expression patterns of *Etv1,* and *Etv5* in rats.

TF-rPGCLCs	No. of testes transplanted	No. of testes with successful transfer	No. of Testes with EGFP positive tubules (%)	No. of EGFP positive seminiferous tubules
T-rPGCLC	6	5/6 (83)	5/5 (100)	3, 4, 2, 2, 5
P14BA-rPGCLC	8	7/8 (88)	7/7 (100)	>5, 3, >5, 3, >5, >5, 2
P14BA+E-rPGCLC	5	4/5 (80)	3/4 (75)	>5, 5, 1

Oikawa et al., Table S1

Transplanted TF-rPGCLC	Injected cell type	No. of oocyes survived after injection	No. of oocytes forming pronulei (%)*	No. of cleaved embryos (%)*	No. of embryos transferred	No. of full-term pups (%)**
T-rPGCLC	Spermatid	69	46 (67)	8 (12)	66	3 (5)
P14BA-rPGCLC	Spermatid	94	65 (69)	9 (10)	88	4 (5)
P14BA+E-rPGCLC	Spermatid	125	79 (63)	4 (3)	114	5 (4)

* Percentages for forming pronuclei and first cleaved embryos were calculated from the number of oocytes survived after injection.

** Percentages for full-term pups were calculated from the number of embryos transferred.

Oikawa et al., Table S2

Targeted genes	Primer ID	Sequence
rCdx2	MO231_rCdx2_F	ACAGAAAGCTGGATTGACCGA
	MO232_rCdx2_R	TCACACGATGGTCCCTGAAC
rTbxt	MO186_rTbxt_F	ATGTCCTCCCTTGTCGCTTTAG
	MO187_rTbxt_R	CGGTTCAGTTACAATCCGCTG
rBlimp1	MO095_rPrdm1_For	AGGATGTGGACTGGGTGGAC
	MO096_rPrdm1_Rev	CTTCACGGAACCGGAGTTACA
rPrdm14	MO043_rPrdm14_For	TCTCGGATGTGGGAAATTTTTGA
	MO044_rPrdm14_Rev	GGGGAATCGAGCACAGTTGA
rActb	MO061_rActb_For	CCCGCGAGTACAACCTTCTT
	MO062_rActb_Rev	CGACGAGCGCAGCGATA

Oikawa et al., Table S3

Antibody/Lectin	Company	Cat No.	RRID	Dilution
anti-GFP	Abcam	ab13970	AB_300798	1:500
anti-TFAP2C	SantaCruz	sc-8977	AB_2286995	1:250
anti-OCT3/4	SantaCruz	sc-5279	AB_628051	1:250
anti-DsRed	Takara Bio Inc.	632496	AB_10013483	1:500
anti-mCherry	EnCor	CPCA-mCherry	AB_2572308	1:250
anti-SOX9	Abcam	ab185966	AB_2728660	1:250
anti-PNA	Vector	RL-1072	AB_2336642	1:300

Oikawa et al., Table S4

SUPPLEMENTARY TABLE TITLES

Table S1 Efficiency of spermatogenesis after transplantation of TF-rPGCLCs into seminiferous tubules of neonatal *Prdm14* KO testis, related to Figure 1, 2, and 4

Table S2 Development of embryos fertilized with spermatid derived from TF-rPGCLCs, related to Figure 1, 2, and 4

Table S3 Primer sequences for RT-qPCR, related to Figure S1 and S3

Table S4 Antibodies used for IF, related to Figure 1, 2, and Figure S1

SUPPLEMENTAL METHODS

Animals

Crlj:WI (RGD ID: 2312504) rats were purchased from Charles River Laboratories Japan, Inc. (Kanagawa, Japan). Slc:SD (RGD ID: 12910483) rats were purchased from SLC Japan (Shizuoka, Japan). All experiments were performed in accordance with the animal care and use committee guidelines of the National Institutes of Natural Sciences and University of Tokyo.

Transplantation of the rPGCLCs into seminiferous tubules

About 60-120 of rPGCLC aggregates were prepared for one experiment. Aggregates at day 3 after induction of rPGCLC were dissociated with 500 µl of 0.25% Trypsin-EDTA at 37 ℃ for 5 min. The reaction was terminated by adding 2 ml of DMEM containing 10% FBS, 1% Glutamax and 1% penicillin/streptomycin. FACS-sorted N3T(+) d3 rPGCLCs were collected and suspended into rPGCLC medium at the concentration of $1x10^4$ cells per 2 µl. 0.5 µl of Trypan blue was added (total ~2.5 µl) to the cell suspensions to confirm the successful injection into the efferent duct, visually. For the recipient, genotyped day 5-7 *Prdm14*$^{H2BVenus/mut}$ (KO) neonatal rats were anesthetized with Isoflurane. A glass capillary filled with 2.5 µl of rPGCLC suspensions was carefully punctured into efferent duct of the testis and injected the cell suspensions using FemtoJet injection system (Eppendorf, Hamburg, Germany). After injection, neonatal rats that underwent surgery were kept on the 37 ℃ warming plate for at least 30 min and returned to the mother rats.

Round spermatid injection

Round spermatids were collected from EGFP positive seminiferous tubules. Cells were dissociated and suspended in GL-PBS (Dulbecco's PBS supplemented with 5.6 mM glucose, 5.4 mM sodium lactate and 0.01% polyvinylpyrrolidone) at 4˚C. Round spermatids were selected based on their appearance or collected with the SH800 cell sorter (SONY, Tokyo, Japan) according to a published protocol [35]. Metaphase II stage oocytes were collected in HEPES-R1ECM medium from superovulated Slc:SD rats and denuded the cumulus with 0.1% hyaluronidase. Oocytes were activated with 5 mM ionomycin for 5 min, and incubated for 40 min in mR1ECM medium until injection. ROSI embryos were treated for 4 h with 5 µg/mL cycloheximide (Sigma-Aldrich) in mR1ECM. Embryos were washed with mR1ECM and cultured in a humidified atmosphere of 5% CO_2 at 37 ℃. On the next day, both 2-cell and 2PN formed embryos were transferred into an oviduct of 0.5 dpc pseudo-pregnant Crlj:WI rats. On 21.5 dpc, the recipient females were subjected to a Caesarean section.

Fluorescence-activated cell sorting (FACS)

Cells were dissociated with 0.25% Trypsin-EDTA at 37 ℃ for 5 min. The reaction was terminated by adding 10X volumes of DMEM containing 10% FBS, 1% Glutamax and 1% penicillin-streptomycin. Non-dissociated cells were removed by 70 µm cell strainer then resuspended into FACS buffer (3% FBS in PBS or 0.1% BSA in PBS). The cells were analyzed or sorted using the flow cytometers (SH800 and MA900; SONY or Aria III; BD Biosciences). FACS data were re-

analyzed by Flowjo software (BD Biosciences)

Quantitative reverse transcription PCR

Total RNA was extracted using PicoPure® RNA Isolation Kit and cDNA was synthesized using QuantiTect Reverse Transcription Kit (QIAGEN, Venlo, The Netherland) according to the manufacturer's protocols. RT-qPCR were performed and analyzed as described previously and the primers sequences used in the paper are listed in **Table S3**.

Immunofluorescence analysis

Samples were fixed with 4% paraformaldehyde for 10-30 min at RT or from 4 h to overnight at 4 ℃ depending on the experiments. For making cryosections, samples were treated with a gradient of 10%, 20%, and 30% sucrose and then embedded in OCT compound (Sakura Finetek, Tokyo, Japan). Samples were cut into 7 µm-thick cryosections using Cryostat (Leica Biosystems, Wetzlar, Germany). After air drying, the sections were washed with PBS, PBS with 0.1% Triton X and then incubated with blocking buffer: 5% normal donkey serum (Sigma-Aldrich), 1% BSA, 0.1% Triton X in PBS. Sections were incubated with primary antibodies for 1-2 h at RT or overnight at 4 ℃. After washing with PBS with 0.1% Triton X, the sections were incubated with fluorescent-conjugated secondary antibodies with DAPI (Dojindo) for 1 h at RT or overnight at 4 ℃. After washing with 0.1% Triton X in PBS, samples were mounted with mounting medium. At least 3 sections were analyzed for each condition. Antibodies used are listed on **Table S4**. Specimens were observed and analyzed using FV3000

(Olympus; Olympus, Tokyo, Japan).

Preparation of RNA-sequencing libraries

Total RNA was extracted using the PicoPure RNA Isolation Kit following the manufacturer's protocol. cDNA library was constructed using SMART-Seq v4 Ultra Low Input RNA Kit (Takara Bio) or SMART-Seq HT PLUS Kit (Takara Bio) and following the manufacturer's recommendations. For Illumina sequencing, cDNA was synthesized from 1 ng of total RNA with 10-15 cycles of PCR amplification, subsequently, 5 ng of cDNA was used for addition of Illumina's adaptors with 13-15 cycles of PCR amplification. The quality and quantity of RNA-seq libraries were evaluated by qPCR using KAPA Library Quantification Kit (Kapa Biosystems). All libraries were pooled and applied to single-end 86 bp sequencing on NextSeq 500 system (Illumina, San Diego, CA) using High Output Kit v2.5. Basecalls were performed using NextSeq 500/550 RTA software (v2.11.3). FASTQ files were generated using bcl2fastq (v2.17.1.14). Two technical replicates of all samples were used for the analysis. RNA-seq data had been deposited in the Sequence Read Archive (SRA) under BioProject ID: PRJNA1199573.

Bioinformatics analysis

For processing the RNA-seq data, we used RaNA-seq program (https://ranaseq.eu/index.php) to calculate TPM values. For further analysis, rat genes showing maximum log2 (TPM+1) values >4 in at least one replicate were selected (9133 genes). Hierarchical clustering was performed based on Ward's

method using the ‘htclust’ function of R package. The PCA was performed using the ‘tidyverse’ function of the R package.

Declaration of generative AI and AI-assisted technologies in the writing process

During the preparation of this work the author(s) used ChatGPT in order to improve refining language and grammar. After using this tool, the authors reviewed and edited the content as needed and take full responsibility for the content of the publication.
